# π–π Noncovalent Interaction Involving 1,2,4- and 1,3,4-Oxadiazole Systems: The Combined Experimental, Theoretical, and Database Study

**DOI:** 10.3390/molecules26185672

**Published:** 2021-09-18

**Authors:** Sergey V. Baykov, Alexander S. Mikherdov, Alexander S. Novikov, Kirill K. Geyl, Marina V. Tarasenko, Maxim A. Gureev, Vadim P. Boyarskiy

**Affiliations:** 1 Institute of Chemistry, Saint Petersburg State University, 7/9 Universitetskaya Nab., 199034 Saint Petersburg, Russia; s.baykov@spbu.ru (S.V.B.); asm93@yandex.ru (A.S.M.); ja2-88@mail.ru (A.S.N.); kirillgeyl@outlook.com (K.K.G.); 2 Pharmaceutical Technology Transfer Centre, Yaroslavl State Pedagogical University Named after K.D. Ushinsky, 108 Respublikanskaya St., 150000 Yaroslavl, Russia; mkarunnaya@mail.ru; 3 Research Center “Digital Biodesign and Personalized Healthcare”, I.M. Sechenov First Moscow State Medical University, 119991 Moscow, Russia; gureev_m_a@staff.sechenov.ru

**Keywords:** noncovalent interactions, π···π interactions, oxadiazoles, X-ray diffraction studies, computational studies, databases search

## Abstract

A series of *N*-pyridyl ureas bearing 1,2,4- (**1a**, **2a**, and **3a**) and 1,3,4-oxadiazole moiety (**1b**, **2b**, **3b**) was prepared and characterized by HRMS, ^1^H and ^13^C NMR spectroscopy, as well as X-ray diffraction. The inspection of the crystal structures of (**1**–**3**)**a**,**b** and the Hirshfeld surface analysis made possible the recognition of the (oxadiazole)···(pyridine) and (oxadiazole)···(oxadiazole) interactions. The presence of these interactions was confirmed theoretically by DFT calculations, including NCI analysis for experimentally determined crystal structures as well as QTAIM analysis for optimized equilibrium structures. The preformed database survey allowed the verification of additional examples of relevant (oxadiazole)···π interactions both in Cambridge Structural Database and in Protein Data Bank, including the cocrystal of commercial anti-HIV drug Raltegravir.

## 1. Introduction

Intermolecular interactions involving aromatic rings, primarily arene stacking, impact ligand–protein binding [1,2], the folding of macromolecules [3,4], the solid-state packing of light-emitting and high energetic materials [5,6,7], as well as the productivity of organocatalysts [8,9]. Therefore, the understanding nature and factors that affect the strength of these interactions could help to utilize them for the design of novel materials and pharmaceuticals.

Stacking interactions between heterocycles and the aromatic amino acid side chains Phe, Tyr, and Trp are topics of interest [10,11] in the context of medicinal chemistry due to the prevalence of heterocyclic scaffolds among marketed drugs [12,13,14,15,16,17]. Although modern experimental studies, together with high accuracy quantum chemical calculations [18,19,20,21,22], significantly shed light on the area of heterocycle stacking, this field is not fully elucidated and there are many information gaps.

To date, such interactions have not been comprehensively investigated for oxadiazoles. This is surprising, since oxadiazole core is one of the privileged structural motifs for medicinal chemistry [23,24], because it combines the favorable ADME profile with low toxicity. Among all the four oxadiazole types, 1,2,4- and 1,3,4-isomers are the most investigated for medicinal applications [23], and they are present in numerous marketed drugs, such as the Ataluren [25], Azilsartan [26], Opicapone [27], Naldemedine [28], and Raltegravir [29]. Moreover, many oxadiazole derivatives have been studied as antibiotics [30,31,32,33,34], fungicides [35], antivirals [36], anticancer [37,38,39], and anti-inflammatory [40,41,42] agents, neuroprotectors [43,44,45], as well as antidiabetic drugs [46] in recent years. This significantly stimulates further structural and theoretical studies on various noncovalent interactions involving oxadiazole-based compounds.

Previously, Freccero and co-workers studied the binding of oxadiazole–pyridine hybrids with G-quadruplex DNA by resonance energy transfer melting assays, fluorescent intercalator displacement assay, and circular dichroism spectroscopy [47,48]. According to their assumption, the π···π stacking interactions can make a significant contribution in this process. Additionally, the stacking interactions were suggested as a part of Ataluren (3-[5-(2-fluorophenyl)-1,2,4-oxadiazol-3-yl]benzoic acid) binding mode toward the potential target (mutated mRNA) by molecular dynamics technique [49]. However, the role of oxadiazole moiety in these processes is still unclear. 

Only recently, the involvement of oxadiazole core in stacking-like intermolecular interactions was demonstrated based on structural and theoretical data of two 1,3,4-oxadiazole derivatives [50]. The performed docking studies of binding these compounds to recombinant human acetylcholinesterase also demonstrated the possibility of stacking interactions between oxadiazole ring and aryl moiety of tyrosine, tryptophan, and phenylalanine in the binding site.

**Figure 1:**
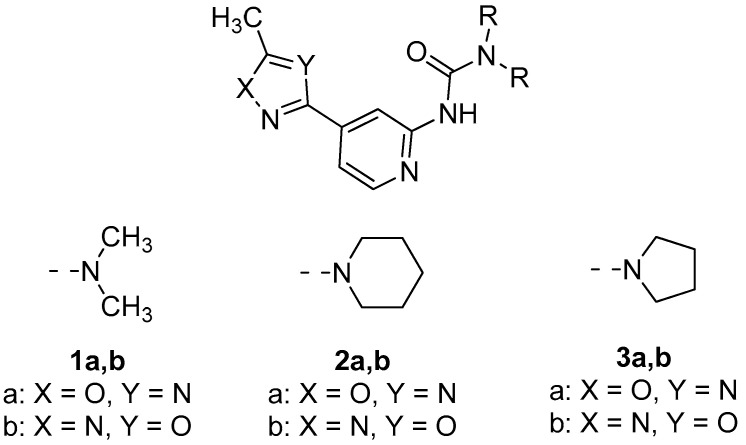
*N*-Pyridyl ureas bearing oxadiazole moiety considered in this study.

In this work, we aimed to achieve a better understanding of the nature of the stacking interactions involving oxadiazole moieties and their possible effects on both molecular and protein binding. Recently, we have already described the synthesis of *N*-pyridyl ureas bearing oxadiazole moiety [51]. Using the suggested synthetic strategy, we obtained and crystalized the representative set of 6 1,2,4-(**1**–**3a**), and 1,3,4-oxadiazole (**1**–**3b**) based *N*-pyridyl ureas (Figure 1). The careful inspection of the collected single-crystal X-ray diffraction (XRD) data on these compounds showed that oxadiazole cores are involved in the π···π/lp···π noncovalent interactions involving pyridine and oxadiazole moieties. The presence of these interactions was confirmed theoretically by DFT calculations followed by the NCI analysis in the XRD structures and by the topological analysis of the electron density distribution within the framework of Bader’s theory (QTAIM method) for the optimized supramolecular associates. Moreover, we have also additionally processed the Cambridge Structural Database and Protein Data Bank on the presence of relevant (oxadiazole)···π interactions in the crystal structures of small molecules as well as their complexes with proteins. These results are discussed below.

## 2. Results and Discussion

### 2.1. General Consideration of XRD Structures of *(**1**–**3**)**a**,**b***

Compounds (**1**–**2**)**a**,**b** were crystallized from chloroform, whereas for the crystallization of the **3a**,**b** a 1,2-DCE solution was used. For **1a**,**b** and **2a**,**b**, the 1,2,4- and 1,3,4-oxadiazole species are isostructural in pairs and crystallize in the *C2/c* and *P2*_1_*/c* space groups, respectively, whereas **3a** and **3b** crystallize from differently: **3a** gives monohydrate (*P2*_1_*/c* space group), while **3b** form crystals as an individual compound (*P2*_1_*/n* space group). The crystal data, data collection parameters, and structure refinement data for (**1**–**3**)**a**,**b** are given in Tables S1 and S2; the plots of structures with “all-atom-numbering” are shown in Figures S1–S6 (Supplementary Information).

### 2.2. Hirshfeld Surface Analyses of *(**1**–**3**)**a**,**b***

**Figure 2:**
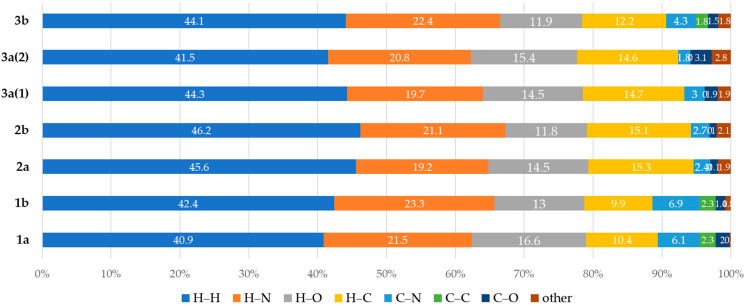
Contributions of various intermolecular contacts to the molecular Hirshfeld surfaces of (**1**–**3**)**a**,**b**.

**Figure 3:**
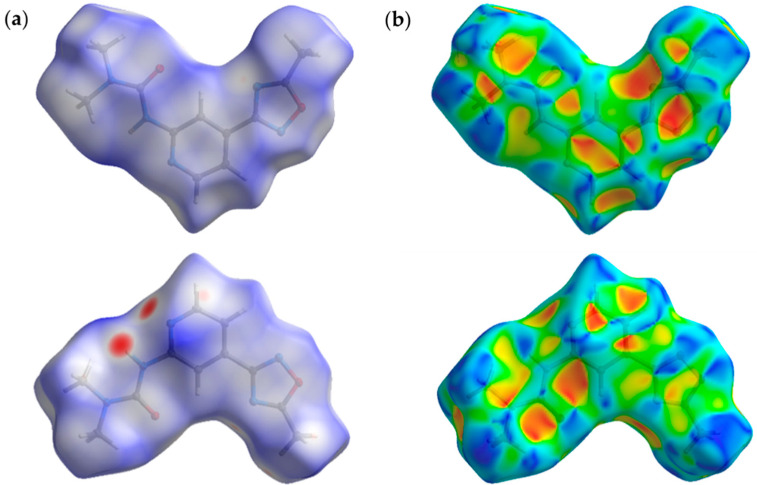
The Hirshfeld surfaces for **1a**, mapped with *d*_norm_ over the range –0,20 (red) to 1,00 (blue) (**a**) moieties; shape index (S), mapped from −1,0 (concave hollows; red) → 0,0 (minimal saddle; green) → +1,0 (convex bumps; blue) (**b**).

We carried out the Hirshfeld surface analyses (HSA) [52,53,54] for the XRD structures of (**1**–**3**)**a**,**b** and systematized types of short contacts to verify what kind of intermolecular forces contribute to the crystal packing (Figure 2). The molecular Hirshfeld surface represents an area where molecules come into contact, and its analysis gives the possibility of additional insight into the nature of intermolecular interactions in the crystal state. For the visualization, we used a mapping of the normalized contact distance (*d*_norm_) [55] and the shape index (Figure 3a,b). HSA for all XRD structures indicates the domination of the contacts involving hydrogen atoms, viz. H–H, H–N, and H–C. However, these contacts provide the largest contributions to the molecular Hirshfeld surfaces because the fraction of these atoms is maximal, and HSA does not disclose the attractive or repulsive nature of these contacts. 

### 2.3. Noncovalent Bonding Patterns

**Figure 4:**
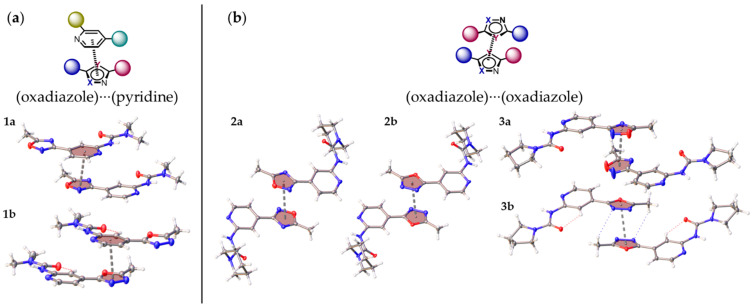
(Oxadiazole)···(pyridine) (**a**) or (oxadiazole)···(oxadiazole) (**b**) interactions in the XRD structure of (**1**–**3**)**a**,**b**.

**Figure 5:**
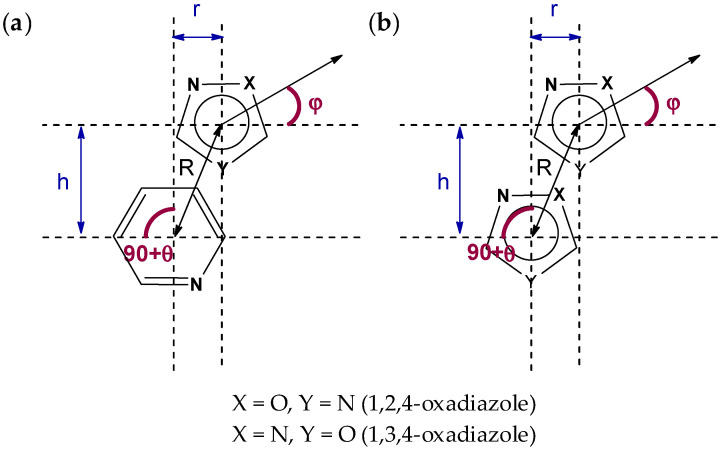
Characteristic parameters of the π···π interactions between oxadiazole and pyridine rings (**a**) and oxadiazoles rings (**b**).

**Figure 6:**
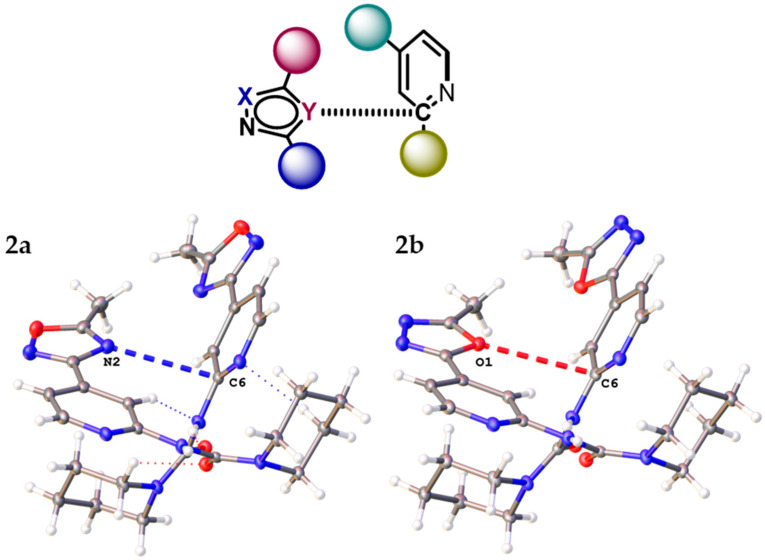
lp–π (oxadiazole)···(pyridine) interaction in the XRD structures of **2a**,**b**.

The further consideration of the HSA data suggests the existence of several types of intermolecular noncovalent contacts in structures of (**1**–**3**)**a**,**b** besides multiple hydrogen bonds. All structures display nontrivial interactions involving 1,2,4- or 1,3,4- oxadiazole core. Depending on the dialkyl substituents at urea moieties in (**1**–**3**)**a**,**b**, the XRD structures display either (oxadiazole)···(pyridine) (Figure 4a) or (oxadiazole)···(oxadiazole) (Figure 4b) interaction. All these interactions could be identified as π–π interactions according to their distance and angular parameters (Table 1, Figure 5). The structures of **1a**,**b** with dimethyl substituted urea display π···π (oxadiazole)···(pyridine) interactions, whereas **2a**,**b** are prone to form (oxadiazole)···(oxadiazole) interactions. In addition, the lp–π interactions involving oxadiazole moiety were found in **2a**, between N2A(oxa)···C1(oxa) (*d* = 3.288(2) Å) and in **2b**, between O1(oxa)···C6(oxa) (3.195(1) Å) (Figure 6).

### 2.4. Theoretical Study of Noncovalent Interactions in *(**1**–**3**)**a**,**b***

**Figure 7:**
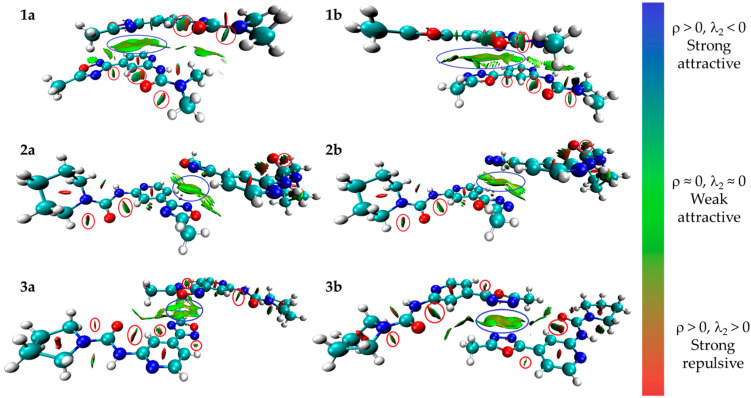
Visualization of various noncovalent interactions involving oxadiazole moieties in 3D using an NCI analysis technique for dimeric associates of (**1**–**3**)**a,b**.

Inspection of the crystallographic data of (**1**–**3**)**a**,**b** suggests the presence of various noncovalent interactions involving oxadiazole moieties in all studied cases. In order to confirm or deny the hypothesis on the existence of these supramolecular contacts, we carried out DFT calculations at the M06-2X/6-31++G** level of theory and the NCI analysis [56] for model dimeric associates of (**1**–**3**)**a**,**b** represented in Figure 4 (see XYZ-files in Supplementary File S2). The NCI analysis based on promolecular density is presented as the visualization of various noncovalent interactions in 3D, using the NCI analysis technique for model supramolecular associates (**1**–**3**)**a,b** in Figure 7 and Figure S10, as a scatter graph of reduced density gradient (RDG) vs. real space function sign(λ_2_)ρ, namely the product of sign of λ_2_ (second largest eigenvalue of Hessian matrix of electron density) and ρ (electron density) (NCI plots [57]). The overall patterns of 2D NCI plots are very similar in all cases. The analysis of 3D NCI plots indicates the presence of a dispersive interaction region (ρ, λ_2_ ≈ 0; the green regions indicated by blue ellipses) in the space between oxadiazole and pyridine rings in **1a,b,** and between oxadiazole rings in (**2**–**3**)**a,b**, which is typical for π–π stacking interactions [58,59,60]. Additionally, several regions with attractive interactions (ρ > 0, λ_2_ < 0; indicated by red ellipses) corresponding to intramolecular hydrogen bonds were found in all model structures.

In addition, we also performed the geometry optimization procedure for all model dimeric associates of (**1**–**3**)**a,b** at the ωB97XD/6-31G* level of theory. It was found that the geometries of the model supramolecular associate of **1a** and **1b** were not significantly changed during the geometry optimization (Figures S11 and S12) and the found (oxadiazole)···(pyridine) interactions are present both in experimental XRD and optimized structures. The latter was confirmed for the optimized structures by the performed topological analysis of the electron density distribution within the framework of Bader’s theory (QTAIM method) (Figures S13 and S14) [61], and the π···π interactions were referred based on bond paths involving any atoms of both rings and appropriated bond critical points (3, −1) (BCPs). At the same time, the optimization procedure in the case of dimeric associates of (**2**–**3**)**a,b** leads to more dramatic changes (Figure S15, Table S3). In all cases, the optimization preserves the dimeric structure of all associates; however, the (oxadiazole)···(oxadiazole) interactions observed in the XRD structures disappear. Instead, all the optimized structures of (**2**–**3**)**a,b** display the presence of appropriate BCPs for the C···C, C···N and N···N contacts corresponding to the (oxadiazole)···(pyridine) interaction according to the results of QTAIM analysis (Figures S16–S19, Table S4). In all dimeric structures of (**1**–**3**)**a,b**, the low magnitude of the electron density (0.003–0.007 a.u.), positive values of the Laplacian (0.008–0.019 a.u.), and zero or very close to zero positive energy density in the found BCPs are typical for noncovalent interactions. The balance between the Lagrangian kinetic energy G(**r**) and potential energy density V(**r**) at the BCPs reveals that a covalent contribution is absent in all supramolecular contacts [62]. The results of QTAIM analysis for the optimized structures of the dimers of (**1**–**3**)**a,b** are summarized in Table S3 (Supplementary Materials), while its Cartesian atomic coordinates are given as XYZ-files in Supplementary File S2. 

Thus, the results of the geometry optimization procedure indicate that (**1**–**3**)**a,b** are prone to form supramolecular associate linked by π···π interactions involving an oxadiazole core. Although the dramatical changes in the geometry of (**2**–**3**)**a,b** presume the greater favorability of the (oxadiazole)···(pyridine) interactions over the (oxadiazole)···(oxadiazole) interactions, the very fact of the participation of the oxadiazole π-system in the π···π interaction is beyond doubt.

### 2.5. Supramolecular Association in Solution

The obtained theoretical results indicate that (**1**–**3**)**a,b** could be prone to supramolecular association not only in a solid state but also in a solution. Therefore, we decided to study this phenomenon by performing a series of NMR measurements of diffusion coefficients (*D*) in CDCl_3_ for model 1,2,4-oxadiazole species **1a** in the concentration range from 6 to 611 mM (for details, see Section S4, Supplementary Materials). The observed diffusion coefficient (*D_obs_*) is an average of the species excising in a solution weighted with their relative amount and is sensitive to the effective size of supramolecular associates [63,64,65,66,67]. To eliminate the effect of changing viscosity at various concentrations on the *D_obs_*, tetramethylsilane (TMS) was used as an internal standard. To estimate the degree of association in the solution we have used the aggregation number *N* parameter. The latter is defined as (*D_o_*/*D_obs_*)^3^, where *D_o_* corresponds to the formal absence of association upon infinite dilution. The aggregation number (*N*) at the highest studied concentration was found to be 1.42 (at 611 mM), indicating the presence of weak association in the solution (Table S5). However, the further consideration of ^1^H and ^13^C NMR data of **1a** at various concentrations has shown that the signals of NH proton and the pyridine and urea carbon atoms are most affected by the variation in concentration, while almost no change for ^13^C chemical shifts of carbons in the oxadiazole ring was observed. Taking this into account, we can conclude that most likely the association should be more driven by the formation of hydrogen bonds rather than by the π···π interactions (Table S6).

### 2.6. CSD and PDB Search of (Oxadiazole)···π Interaction

Our Cambridge Structure Database (CSD, version 5.41) survey verified approximately 406 and 658 structures with uncomplexed 1,2,4- and 1,3,4-oxadiazoles, respectively. We have processed these structures on the presence of oxadiazole···π interaction involving 6- and 5-membered aromatic and heteroaromatic π-systems. The analysis of the CSD data on 1,2,4-oxadiazoles species indicates 74 structures with intermolecular interaction involving six-membered rings and 76 entities involving five-membered rings. Noteworthy, that 58 contacts involving five-membered π-systems correspond to homoleptic (oxadiazole)···(oxadiazole) interaction similar to those found in the structures (**2**–**3**)**a**,**b**. In the case of 1,3,4-oxadiazoles, 233 structures with intermolecular interactions involving six-membered aromatic and heteroaromatic π-systems were found, whereas five-membered π-systems form these contacts in only 97 cases. Similar to 1,2,4-oxadiazole species, most of the latter interactions belong to (oxadiazole)···(oxadiazole) contacts (76 structures). 

Moreover, we also separately analyzed the participation of C, N, and O atoms of 1,2,4- and 1,3,4-oxadiazole rings in π···π contacts found in the CSD (Table 2). The obtained data shows both carbon atoms in oxadiazoles are participate in the π···π interaction almost in all found cases. The contribution of contacts involving oxadiazole nitrogen atoms is noticeably lesser in the case of the π···π interaction involving six-membered π-systems; however, it became more significant in case of interaction with five-membered rings and (oxadiazole)···(oxadiazole) contacts.

Remarkably, that our CSD search also verified the studied (oxadiazole)···π interactions in the crystal structures of two 1,3,4-oxadiazole based biologically active compounds: Raltegravir (CCDC DIRCIS) and Zibotentan (CCDC OCAGAB). Raltegravir is the first integrase inhibitor, which was approved by FDA for the HIV treatment [68]. It has been marketed by Merck&Co under the brand name Isentress™ and its annual sales for 2019 were USD 975 million [69]. Zibotentan, also known as ZD4054, is an experimental anticancer agent involved in several clinical and preclinical studies [70,71].

**Figure 8:**
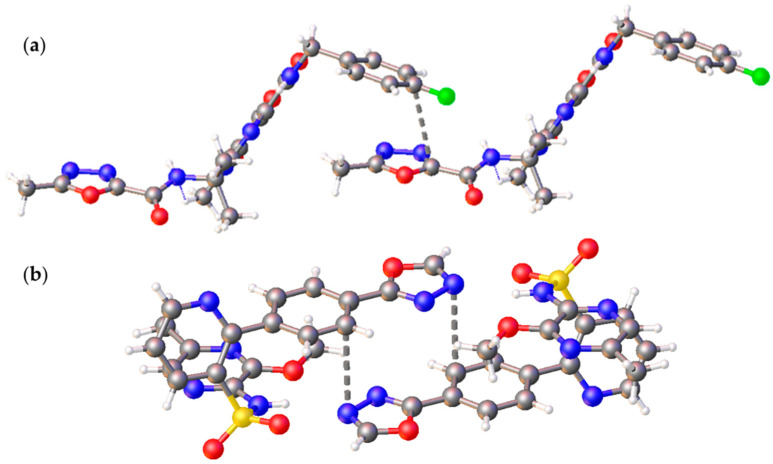
The (oxadiazole)···π interactions found in the crystal structures of Raltegravir (CCDC DIRCIS) (**a**); and Zibotentan (CCDC OCAGAB) (**b**).

Both crystal structures display the intermolecular short (oxadiazole)···(arene) contacts between carbon atoms (3.563 Å) in the case of Raltegravir (**CCDC** DIRCIS) and between arene carbon and oxadiazole nitrogen (3.314 Å) for Zibotentan (**CCDC** OCAGAB) (Figure 8).

**Figure 9:**
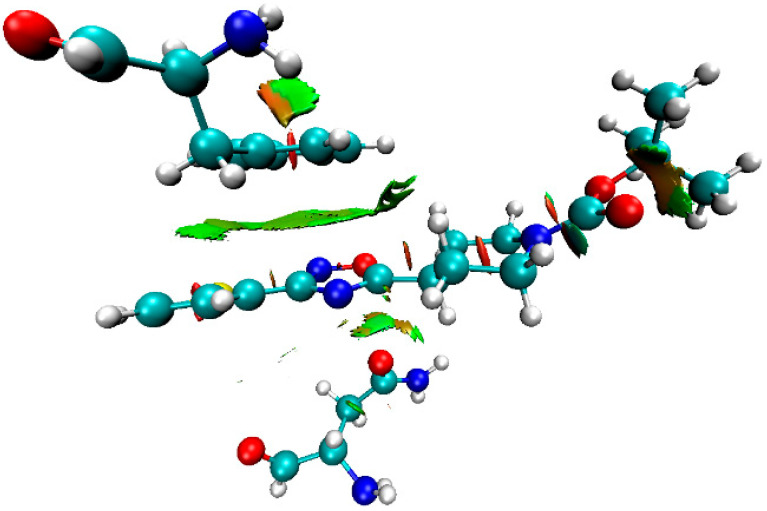
Visualization of (oxadiazole)···π and (oxadiazole)···lp interactions in 3D using NCI analysis technique for the fragment of 3GO1 PDB structure.

In addition, the application of the IsoStar 2020.3 package allowed the verification of eight cases of (1,2,4-oxadiazole)···phenyl interaction in the PDB database. Among the eight structures of protein complexes with 1,2,4-oxadiazole based small molecules found, only four could be considered as the (oxadiazole)···π interaction based on their distance and angular parameters: 3GO1, 3IES, 5MAR, and 6BTN. The shortest (oxadiazole)···π interaction was found in case of the complex of 1,2,4-oxadiazole based anti-HIV-1 Fab 268-D species with V3 peptide MN [72] (PDB code: 3GO1), in which 1,2,4-oxadiazole moiety is in spatial proximity with the phenyl group from the phenylalanine fragment (shortest contact C···N 3.422 Å). The conducted NCI analysis for the reduced model cluster of 3GO1 (Figure 9, see Section S3 for the full model cluster preparation details, Supplementary Materials) confirmed the existence of this (oxadiazole)···π interaction. Additionally, the NCI analysis also verified one more noncovalent interaction involving the oxadiazole ring and the lone pair of an amide oxygen atom (2.834 Å). 

The found 3GO1 structure demonstrated that the discussed π-interactions are involved in ligand–protein binding and that their consideration is necessary for a clearer understanding of pharmacological processes. Particularly, such data allow improved docking scoring functions and other algorithms applied for computer-aided drug discovery [73,74].

## 3. Material and Methods

### 3.1. General

*N*-Oxides were synthesized according to literature procedures [51]. All other reactants and solvents were obtained from commercial sources. ^1^H (400 MHz) and ^13^C (101 MHz) NMR spectra, as well as diffusion coefficient measurements) were recorded on a Bruker AVANCE III 400 spectrometer at room temperature (RT) using CDCl_3_ or DMSO-*d*_6_ as solvents. The chemical shifts (δ) are given in ppm and referenced to the residual signals of solvents: 2.50 ppm for residual ^1^H, 39.50 ppm for ^13^C in DMSO–*d*_6_; and 7.26 ppm for residual ^1^H, 77.16 ppm for ^13^C in CDCl_3_. Multiplicities are abbreviated as follows: s = singlet, d = doublet, t = triplet, q = quartet, m = multiplet, br = broad; coupling constants, *J*, are reported in Hertz (Hz). Melting points were determined in open capillary tubes on Electrothermal IA 9300 series Digital Melting Point Apparatus. High-resolution mass spectra (HRMS) were measured on Bruker Maxis HRMS-ESI-qTOF (Electrospray Ionization, ESI).

### 3.2. Oxadiazoles Preparation and Characterization

The preparation and characterization of compounds **1a**, **2a**, and **2b** were described by us previously [51]. Other oxadiazoles (**1b**, **3a**, and **3b**) were synthesized via the acid-catalyzed reaction of corresponding pyridine-*N*-oxides with dialkylcyanamides according to the described protocol [51]. A mixture of the substituted pyridine-*N*-oxide (1 mmol), the dialkylcyanamide (2.0 mmol) and acetonitrile (1 mL, 10.0 mmol) was stirred at room temperature for 2 min, and then MsOH (144 mg, 1.5 mmol) was added dropwise within 3 min. The reaction mixture was stirred at 60 °C for 3 h, cooled, diluted with saturated aq. Na_2_CO_3_ (2 mL) and aq. NaCl (5 mL), and extracted with EtOAc (4 × 10 mL). Combined organic fractions were dried with anhydrous Na_2_SO_4_, filtered, and concentrated on a rotary evaporator. The crude product was purified by column chromatography on silica gel (EtOAc/hexane (2:1) for 1,2,4-oxadiazoles and acetone/DCM (1:19) for 1,3,4-oxadiazoles) to give the target compound.

**1,1-Dimethyl-3-(4-(5-methyl-1,3,4-oxadiazol-2-yl)pyridin-2-yl)urea 1b**

Beige solid, 65% (161 mg) yield, m.p. 193–195 °C. ^1^H NMR (400 MHz, DMSO-*d*_6_): δ 9.21 (s, 1H), 8.44 (dd, *J* = 5.2, 0.8 Hz, 1H), 8.42–7.40 (br s, 1H), 7.49 (dd, *J* = 5.2, 1.5 Hz, 1H), 2.98 (s, 6H), 2.62 (s, 3H). ^13^C NMR (101 MHz, DMSO-*d*_6_): δ 165.3, 163.3, 155.5, 155.3, 149.4, 132.3, 114.2, 109.7, 36.7, 11.1. HRMS (ESI), *m/z* [M + H]^+^ calcd for C_11_H_13_N_5_O_2_: 248.1142; found: 248.1139.

***N*-(4-(5-Methyl-1,2,4-oxadiazol-3-yl)pyridin-2-yl)pyrrolidine-1-carboxamide 3a**

Beige solid, 66% (180 mg) yield, m.p. 102–104 °C. ^1^H NMR (400 MHz, CDCl_3_): δ = 8.99 (s, 1H), 8.27 (d, *J* = 5.6 Hz, 1H), 8.22–8.06 (br s, 1H), 7.63 (dd, *J* = 5.6, 1.2 Hz, 1H), 3.58 (m, 4H), 2.68 (s, 3H), 2.02 (m, 4H). ^13^C NMR (101 MHz, CDCl_3_): δ 177.2, 167.0, 153.5, 152.6, 146.9, 137.1, 115.6, 111.6, 46.0, 25.6, 12.3. HRMS (ESI), *m/z* [M + H]^+^ calcd for C_13_H_15_N_5_O_2_: 274.1299; found: 274.1327.

***N*-(4-(5-Methyl-1,3,4-oxadiazol-2-yl)pyridin-2-yl)pyrrolidine-1-carboxamide 3b**

Beige solid, 65% (178 mg) yield m.p. 133–135 °C. ^1^H NMR (400 MHz, CDCl_3_): δ = 8.81 (s, 1H), 8.32 (d, *J* = 5.6 Hz, 1H), 8.00–7.75 (br s, 1H), 7.70 (dd, *J* = 5.6, 1.2 Hz, 1H), 3.62–3.52 (m, 4H), 2.65 (s, 3H), 2.07–1.98 (m, 4H). ^13^C NMR (101 MHz, CDCl_3_): δ 164.7, 163.4, 153.6, 152.7, 147.9, 133.2, 115.0, 109.9, 45.9, 25.6, 11.1. HRMS (ESI), *m/z* [M + H]^+^ calcd for C_13_H_15_N_5_O_2_: 274.1299; found: 274.1292.

### 3.3. Crystallography

X-ray diffraction data were collected at 100 K on an Xcalibur Eos diffractometer (for **1a**, **2a**, and **3b**) using Mo-Kα (λ = 0.71073 nm) radiation and a SuperNova diffractometer (for **1b**, **2b**, and **3a**) using Cu-Kα (λ = 0.154184 nm) radiation. All structures have been solved with the SHELX program [75] using intrinsic phasing and refined with the ShelXL program [76] using least squares minimization. Both programs are incorporated in the OLEX2 program package [77]. An empirical absorption correction was applied in the CrysAlisPro [78] program complex using spherical harmonics, implemented in the SCALE3 ABSPACK scaling algorithm. Supplementary crystallographic data for this paper have been deposited at Cambridge Crystallographic Data Centre (CCDC numbers 1903615, 1903620, 2093232, 2093234, 2093238, 2093239) and can be obtained free of charge via www.ccdc.cam.ac.uk/data_request/cif (accessed on 7 September 2021).

### 3.4. Hirshfeld Surface Analysis

The Hirshfeld surface analysis was carried out using the CrystalExplorer 17.5 software [79]. The normalized contact distances, *d*_norm_, based on Bondi’s van der Waals radii, were mapped [55] onto the Hirshfeld surface. In the color scale, negative values of *d*_norm_ are visualized by the red color indicating contacts shorter than the sum of van der Waals radii. The white color denotes intermolecular distances that are close to van der Waals contacts with *d*_norm_ equal to zero. In turn, contacts longer than the sum of the van der Waals radii with positive *d*_norm_ values are colored with blue.

### 3.5. Computational Study

The single point calculations based on the experimental X-ray geometries of (**1**–**3**)**a**,**b** have been carried out at the DFT level of theory using the M06-2X functional [80] and standard 6-311++G** basis sets for all atoms with the help of Gaussian-09 [81] program package. The full geometry optimization of dimeric associates of (**1**–**3**)**a**,**b** as well as DFT calculations for **3GO1** structure (see Section S3 for the model cluster preparation details, Supplementary Materials) were carried out using the dispersion-corrected hybrid functional ωB97XD [82] with 6-31G*((**1**–**3**)**a**,**b** and **3GO1**) and 6-311+ G* (**1a,b**) basis sets for all atoms with the help of Gaussian-09 [81] program package. The topological analysis of the electron density distribution with the help of the atoms in molecules (QTAIM) method developed by Bader [61] and NCI analysis [56] have been performed by using the Multiwfn program (version 3.7) [83]. The VMD program [84] was additionally used for visualization of noncovalent interactions. The Cartesian atomic coordinates of the model structures of (**1**–**3**)**a**,**b** and the model cluster of **3GO1** are given as XYZ-files in Supplementary File S2.

### 3.6. Databases Search Processing

**Figure 10:**
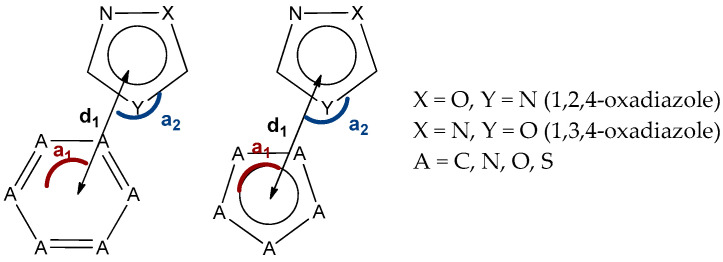
The parameters utilized for processing of the CSD and PDB data.

Processing of the Cambridge Structure Database (CSD; v 5.42) was performed using the ConQuest module (v 2020.3.0), whereas the Protein Data Bake (PDB) database was surveyed with help of IsoStar 2020.3 package. The CSD analysis for the (oxadiazole)···π interactions includes only the interactions involving 6- and 5-membered aromatic and heteroaromatic π-systems, while the PDB survey included only the interactions between 1,2,4-oxadiazole and phenyl moieties. The interaction analysis was based on three parameters: viz. distance C···X (d_1_), and angles (a_1_) and (a_2_) (Figure 10). The distances were restricted by the sum of Bond vdW radii +0.5 Å and a1–2 angularity was restricted to in a range from 65 to 115, whereas X was any atom except hydrogen. In the case of several contacts, the shortest contact was selected. For the CSD search, only structures with determined 3D and with no error were included in the search query. In addition to this, powder structures were excluded from the search. To ensure that we have only high-quality structures, R-factor which represents the agreement between the obtained crystallographic model and the experimental diffraction data was kept below 0.1.

## 4. Concluding Remarks

In this work, we have synthesized and crystalized the representative set of six 1,2,4- (“a” series, Figure 1) and 1,3,4-oxadiazole (“b” series) based *N*-pyridyl ureas, which were studied by single-crystal X-ray diffraction. Inspection of the crystallographic data and the Hirshfeld surface analysis for (**1–3**)**a**,**b** suggests the presence of various noncovalent interactions involving oxadiazole moieties in all studied cases. Depending on the dialkyl substituents at urea moieties in (**1**–**3**)**a**,**b**, the XRD structures display either (oxadiazole)···(pyridine) (**1a**,**b**, Figure 4a) or (oxadiazole)···(oxadiazole) (**2a**,**b**, Figure 4b) interaction, which should be identified as π–π interactions according to their distance and angular parameters. The involvement of the oxadiazole system in π–π interactions was confirmed theoretically by DFT calculations including NCI (for the XRD structures) and QTAIM (for the optimized equilibrium structures of associates) analyses. 

The performed database survey revealed a number of additional examples of relevant (oxadiazole)···π interactions both in the Cambridge Structural Database (CSD) and Protein Data Bank (PDB). The analysis of the CSD data allowed verification of ca. 150 and 330 examples, where 1,2,4-oxadiazoles and 1,3,4-oxadiazoles cores are participating in intermolecular π···π interaction involving six- and five-membered π-systems. Moreover, the studied (oxadiazole)···π interactions were also recognized in the crystal structures of two 1,3,4-oxadiazole based biologically active compounds: Raltegravir (CCDC DIRCIS) and Zibotentan (CCDC OCAGAB). In addition, the application of the IsoStar 2020.3 package allowed the verification of four cases of (1,2,4-oxadiazole)···π interaction in the PDB database. The latter demonstrates that the studied (oxadiazole)···π interactions are involved in ligand–protein binding, and their consideration is necessary for a clearer understanding of pharmacological processes and, consequently, for the accurately predicting medicinal properties of compounds. 

Moreover, the current finding on the π···π interactions involving oxadiazole moieties are important not only for the utilization of medicinal chemistry and drug design. Previously, such oxadiazole-involved stacking interactions were recognized as the properties defining force in several fluorescent materials [85,86,87,88].

## Figures and Tables

**Table 1:** Characteristic parameters of the π···π interactions in structures (**1**–**3**)**a**,**b**.

Structure	Type	R	h_1_, Å	h_2_, Å	r_1_, Å	r_2_, Å	φ, °	θ, °
**1a**	a	3.6230(8)	3.5157(10)	3.4774(10)	0.875(3)	1.017(2)	10.7(2)	3.69(5)
**1b**	a	3.5203(9)	3.4735(11)	3.4031(11)	0.572(3)	0.900(2)	8.5(3)	5.75(6)
**2a**	b	3.6838(12)	3.3492(16)	3.3492(16)	1.534(3)	1.534(3)	0.00(14)	0.00(14)
**2b**	b	3.4848(11)	3.2480(14)	3.2480(14)	1.262(2)	1.262(2)	0.00(19)	0.00(18)
**3a**	b	3.5016(11)	3.2481(15)	3.189(2)	3.4064(11)	0.812(3)	68.1(6)	12.56(8)
**3b**	b	3.3978(9)	3.2675(10)	3.2675(10)	0.9321(19)	0.9321(19)	0.00(19)	0.00(13)

R—distances between the centroid of one ring to the centroid of another ring. h_i_—distances between the centroid of one ring to the plane of another. r_i_—distances between the centroid of one ring and projection of another ring centroid to the first plane (shift). φ—twist angle. θ—the angle between ring planes.

**Table 2:** The (oxadiazole)···π interactions found in the CSD and categorized by the type of interacting atom.

π-System	1,2,4-Oxadiazoles				1,3,4-Oxadiazoles			
	Total	C	N	O	Total	C	N	O
six-membered ring	74	74 (C3 68; C5 53)	22 (N1 16; N4 10)	28	233	229	45	74
five-membered rings	76 (58 oxa-oxa)	76 (C3 66; C5 63)	58 (N1 46; N4 26)	38	97 (76 oxa-oxa)	95	58	14

## Data Availability

Data are contained within the article and supplementary materials. Additionally, CIFs are openly available in www.ccdc.cam.ac.uk/data_request/cif (accessed on 7 September 2021).

## Supplementary Materials

The following are available online. Contains crystallographic information for (**1**–**3**)**a,b** (Tables S1 and S2) and copies of NMR spectra.

## Sample Availability

Samples of the compounds are not available from the authors.
