# Supplementary material for: π–π Noncovalent Interaction Involving 1,2,4- and 1,3,4-Oxadiazole Systems: The Combined Experimental, Theoretical, and Database Study
Source: Molecules. 2021 Sep 18;26(18):5672. doi: 10.3390/molecules26185672 (PMC8466036; doi:10.3390/molecules26185672)

checkCIF/PLATON report

Structure factors have been supplied for datablock(s) 1a, 1b, 2a, 2b, 3a, 3b

THIS REPORT IS FOR GUIDANCE ONLY. IF USED AS PART OF A REVIEW PROCEDURE FOR PUBLICATION, IT SHOULD NOT REPLACE THE EXPERTISE OF AN EXPERIENCED CRYSTALLOGRAPHIC REFEREE.

No syntax errors found.   CIF dictionary   Interpreting this report

Datablock: 1a

```
Bond precision:  C-C = 0.0020 A         Wavelength=0.71073

Cell:           a=20.4216(11)    b=4.8213(2)        c=25.8398(15)
                alpha=90         beta=111.743(7)    gamma=90
Temperature:    100 K

                Calculated                 Reported
Volume          2363.2(2)                  2363.1(2)
Space group     C 2/c                      C 1 2/c 1
Hall group      -C 2yc                     -C 2yc
Moiety formula  C11 H13 N5 O2              C11 H13 N5 O2
Sum formula     C11 H13 N5 O2              C11 H13 N5 O2
Mr              247.26                     247.26
Dx,g cm-3       1.390                      1.390
Z               8                          8
Mu (mm-1)       0.101                      0.101
F000            1040.0                     1040.0
F000'           1040.40
h,k,lmax        26,6,33                    26,6,33
Nref            2576                       2566
Tmin,Tmax       0.980,0.990                0.857,1.000
Tmin'           0.980

Correction method= # Reported T Limits: Tmin=0.857 Tmax=1.000
AbsCorr = MULTI-SCAN

Data completeness= 0.996          Theta(max)= 26.999

R(reflections)= 0.0423( 2343)     wR2(reflections)= 0.1049( 2566)

S = 1.060                  Npar= 171
```

The following ALERTS were generated. Each ALERT has the format
test-name_ALERT_alert-type_alert-level.
Click on the hyperlinks for more details of the test.

Alert level C

```
PLAT906_ALERT_3_C Large K Value in the Analysis of Variance ......      3.001 Check
PLAT911_ALERT_3_C Missing FCF Refl Between Thmin & STh/L=    0.600          7 Report
PLAT913_ALERT_3_C Missing # of Very Strong Reflections in FCF ....          4 Note
```

Alert level G

```
PLAT002_ALERT_2_G Number of Distance or Angle Restraints on AtSite          2 Note
PLAT172_ALERT_4_G The CIF-Embedded .res File Contains DFIX Records          1 Report
PLAT395_ALERT_2_G Deviating  X-O-Y    Angle From 120 for O1             106.5 Degree
PLAT860_ALERT_3_G Number of Least-Squares Restraints .............          1 Note
PLAT870_ALERT_4_G ALERTS Related to Twinning Effects Suppressed ..          ! Info
PLAT883_ALERT_1_G No Info/Value for _atom_sites_solution_primary .     Please Do !
PLAT910_ALERT_3_G Missing # of FCF Reflection(s) Below Theta(Min).          3 Note
PLAT933_ALERT_2_G Number of OMIT Records in Embedded .res File ...          3 Note
PLAT941_ALERT_3_G Average HKL Measurement Multiplicity ...........        3.8 Low
PLAT955_ALERT_1_G Reported (CIF) and Actual (FCF) Lmax Differ by .          1 Units
```

```
   0 ALERT level A = Most likely a serious problem - resolve or explain
   0 ALERT level B = A potentially serious problem, consider carefully
   3 ALERT level C = Check. Ensure it is not caused by an omission or oversight
  10 ALERT level G = General information/check it is not something unexpected

   2 ALERT type 1 CIF construction/syntax error, inconsistent or missing data
   3 ALERT type 2 Indicator that the structure model may be wrong or deficient
   6 ALERT type 3 Indicator that the structure quality may be low
   2 ALERT type 4 Improvement, methodology, query or suggestion
   0 ALERT type 5 Informative message, check
```

Datablock: 1b

```
Bond precision:  C-C = 0.0020 A          Wavelength=1.54184

Cell:            a=20.2667(8)    b=4.8772(2)        c=25.3025(9)
                 alpha=90        beta=109.643(4)    gamma=90
Temperature:     100 K
```

	Calculated	Reported
Volume	2355.47(17)	2355.47(17)
Space group	C 2/c	C 1 2/c 1
Hall group	-C 2yc	-C 2yc
Moiety formula	C11 H13 N5 O2	C11 H13 N5 O2
Sum formula	C11 H13 N5 O2	C11 H13 N5 O2
Mr	247.26	247.26
Dx,g cm-3	1.395	1.395
Z	8	8
Mu (mm-1)	0.839	0.839
F000	1040.0	1040.0
F000’	1043.32	
h,k,lmax	24,6,31	24,6,31
Nref	2319	2308
Tmin,Tmax	0.874,0.912	0.424,1.000
Tmin’	0.874	

Correction method= # Reported T Limits: Tmin=0.424 Tmax=1.000
AbsCorr = MULTI-SCAN

Data completeness= 0.995 Theta(max)= 72.421

R(reflections)= 0.0484(1970) wR2(reflections)= 0.1370(2308)

S = 1.030 Npar= 170

The following ALERTS were generated. Each ALERT has the format
test-name_ALERT_alert-type_alert-level.
Click on the hyperlinks for more details of the test.

Alert level C

PLAT250_ALERT_2_C Large U3/U1 Ratio for Average U(i,j) Tensor .... 2.6 Note

---

Alert level G

PLAT002_ALERT_2_G Number of Distance or Angle Restraints on AtSite 2 Note
PLAT172_ALERT_4_G The CIF-Embedded .res File Contains DFIX Records 1 Report
PLAT398_ALERT_2_G Deviating C-O-C Angle From 120 for O1 102.8 Degree
PLAT860_ALERT_3_G Number of Least-Squares Restraints .... 1 Note
PLAT912_ALERT_4_G Missing # of FCF Reflections Above STh/L= 0.600 11 Note
PLAT933_ALERT_2_G Number of OMIT Records in Embedded .res File ... 3 Note
PLAT955_ALERT_1_G Reported (CIF) and Actual (FCF) Lmax Differ by . 1 Units
PLAT978_ALERT_2_G Number C-C Bonds with Positive Residual Density. 1 Info

---

0 **ALERT level A** = Most likely a serious problem - resolve or explain
0 **ALERT level B** = A potentially serious problem, consider carefully
1 **ALERT level C** = Check. Ensure it is not caused by an omission or oversight
8 **ALERT level G** = General information/check it is not something unexpected

1 ALERT type 1 CIF construction/syntax error, inconsistent or missing data

5 ALERT type 2 Indicator that the structure model may be wrong or deficient
1 ALERT type 3 Indicator that the structure quality may be low
2 ALERT type 4 Improvement, methodology, query or suggestion
0 ALERT type 5 Informative message, check

Datablock: 2a

Bond precision: C-C = 0.0019 A Wavelength=0.71073

Cell:	a=15.4284(8)	b=9.4251(5)	c=9.5049(4)
	alpha=90	beta=91.712(4)	gamma=90
Temperature:	100 K		

	Calculated	Reported
Volume	1381.53(12)	1381.53(12)
Space group	P 21/c	P 1 21/c 1
Hall group	-P 2ybc	-P 2ybc
Moiety formula	C14 H17 N5 O2	C14 H17 N5 O2
Sum formula	C14 H17 N5 O2	C14 H17 N5 O2
Mr	287.33	287.32
Dx,g cm-3	1.381	1.381
Z	4	4
Mu (mm-1)	0.097	0.097
F000	608.0	608.0
F000’	608.23	
h,k,lmax	23,14,14	23,13,14
Nref	4941	4391
Tmin,Tmax	0.985,0.987	0.871,1.000
Tmin’	0.983	

Correction method= # Reported T Limits: Tmin=0.871 Tmax=1.000
AbsCorr = MULTI-SCAN

Data completeness= 0.889 Theta(max)= 32.364

R(reflections)= 0.0528(3429) wR2(reflections)= 0.1151(4391)

S = 1.057 Npar= 195

The following ALERTS were generated. Each ALERT has the format
test-name_ALERT_alert-type_alert-level.
Click on the hyperlinks for more details of the test.

Alert level C

PLAT906_ALERT_3_C Large K Value in the Analysis of Variance ...... 6.995 Check
PLAT911_ALERT_3_C Missing FCF Refl Between Thmin & STh/L= 0.600 9 Report

---

Alert level G

```
PLAT395_ALERT_2_G Deviating  X-O-Y    Angle From 120 for O1           106.4 Degree
PLAT910_ALERT_3_G Missing # of FCF Reflection(s) Below Theta(Min).        3 Note
PLAT912_ALERT_4_G Missing # of FCF Reflections Above STh/L=  0.600      524 Note
PLAT933_ALERT_2_G Number of OMIT Records in Embedded .res File ...        9 Note
PLAT941_ALERT_3_G Average HKL Measurement Multiplicity ...........      2.4 Low
PLAT978_ALERT_2_G Number C-C Bonds with Positive Residual Density.        7 Info
```

```
   0 ALERT level A = Most likely a serious problem - resolve or explain
   0 ALERT level B = A potentially serious problem, consider carefully
   2 ALERT level C = Check. Ensure it is not caused by an omission or oversight
   6 ALERT level G = General information/check it is not something unexpected

   0 ALERT type 1 CIF construction/syntax error, inconsistent or missing data
   3 ALERT type 2 Indicator that the structure model may be wrong or deficient
   4 ALERT type 3 Indicator that the structure quality may be low
   1 ALERT type 4 Improvement, methodology, query or suggestion
   0 ALERT type 5 Informative message, check
```

Datablock: 2b

Bond precision: C-C = 0.0019 A Wavelength=1.54184

Cell:	a=15.1212(2)	b=9.5142(1)	c=9.5236(1)
	alpha=90	beta=91.821(1)	gamma=90
Temperature:	100 K		

	Calculated	Reported
Volume	1369.43(3)	1369.43(3)
Space group	P 21/c	P 1 21/c 1
Hall group	-P 2ybc	-P 2ybc
Moiety formula	C14 H17 N5 O2	C14 H17 N5 O2
Sum formula	C14 H17 N5 O2	C14 H17 N5 O2
Mr	287.33	287.32
Dx,g cm-3	1.394	1.394
Z	4	4
Mu (mm-1)	0.801	0.801
F000	608.0	608.0
F000'	609.87	
h,k,lmax	18,11,11	18,11,11
Nref	2772	2754
Tmin,Tmax	0.891,0.908	0.377,1.000
Tmin'	0.873	

Correction method= # Reported T Limits: Tmin=0.377 Tmax=1.000
AbsCorr = MULTI-SCAN

Data completeness= 0.994 Theta(max)= 73.934

```
R(reflections)= 0.0467( 2567)     wR2(reflections)= 0.1303( 2754)

S = 1.037                   Npar= 195
```

The following ALERTS were generated. Each ALERT has the format
test-name_ALERT_alert-type_alert-level.
Click on the hyperlinks for more details of the test.

Alert level G

```
PLAT398_ALERT_2_G Deviating  C-O-C    Angle From 120 for O1          102.6 Degree
PLAT912_ALERT_4_G Missing # of FCF Reflections Above STh/L=  0.600       18 Note
PLAT933_ALERT_2_G Number of OMIT Records in Embedded .res File ...        1 Note
PLAT941_ALERT_3_G Average HKL Measurement Multiplicity ...........      3.0 Low
PLAT978_ALERT_2_G Number C-C Bonds with Positive Residual Density.        3 Info
```

---

0 **ALERT level A** = Most likely a serious problem - resolve or explain
0 **ALERT level B** = A potentially serious problem, consider carefully
0 **ALERT level C** = Check. Ensure it is not caused by an omission or oversight
5 **ALERT level G** = General information/check it is not something unexpected

0 ALERT type 1 CIF construction/syntax error, inconsistent or missing data
3 ALERT type 2 Indicator that the structure model may be wrong or deficient
1 ALERT type 3 Indicator that the structure quality may be low
1 ALERT type 4 Improvement, methodology, query or suggestion
0 ALERT type 5 Informative message, check

Datablock: 3a

```
Bond precision:  C-C = 0.0020 A          Wavelength=1.54184

Cell:            a=8.4559(2)     b=13.9116(3)      c=23.0116(5)
                 alpha=90        beta=93.125(2)    gamma=90
Temperature:     100 K
```

	Calculated	Reported
Volume	2702.95(11)	2702.95(10)
Space group	P 21/c	P 1 21/c 1
Hall group	-P 2ybc	-P 2ybc
Moiety formula	2(C13 H15 N5 O2), H2 O	2(C13 H15 N5 O2), H2 O
Sum formula	C26 H32 N10 O5	C26 H32 N10 O5
Mr	564.62	564.61
Dx,g cm-3	1.388	1.387
Z	4	4
Mu (mm-1)	0.830	0.830
F000	1192.0	1192.0
F000’	1195.80	
h,k,lmax	10,17,28	10,17,28
Nref	5662	5538
Tmin,Tmax	0.879,0.905	0.827,1.000
Tmin’	0.854	

Correction method= # Reported T Limits: Tmin=0.827 Tmax=1.000
AbsCorr = MULTI-SCAN

Data completeness= 0.978 Theta(max)= 76.102

R(reflections)= 0.0438(4542) wR2(reflections)= 0.1175(5538)

S = 1.039 Npar= 375

The following ALERTS were generated. Each ALERT has the format
test-name_ALERT_alert-type_alert-level.
Click on the hyperlinks for more details of the test.

Alert level C

```
PLAT241_ALERT_2_C High   'MainMol' Ueq as Compared to Neighbors of        N1A Check
PLAT250_ALERT_2_C Large U3/U1 Ratio for Average U(i,j) Tensor ....        2.4 Note
PLAT417_ALERT_2_C Short Inter D-H..H-D       H4AA     ..H3B      .       2.10 Ang.
                                                    x,y,z  =      1_555 Check
PLAT906_ALERT_3_C Large K Value in the Analysis of Variance ......      5.563 Check
PLAT911_ALERT_3_C Missing FCF Refl Between Thmin & STh/L=    0.600         11 Report
```

Alert level G

```
PLAT007_ALERT_5_G Number of Unrefined Donor-H Atoms ..............          4 Report
PLAT395_ALERT_2_G Deviating  X-O-Y    Angle From 120 for O1             106.4 Degree
PLAT395_ALERT_2_G Deviating  X-O-Y    Angle From 120 for O1A            106.8 Degree
PLAT720_ALERT_4_G Number of Unusual/Non-Standard Labels ..........          4 Note
PLAT883_ALERT_1_G No Info/Value for _atom_sites_solution_primary .     Please Do !
PLAT912_ALERT_4_G Missing # of FCF Reflections Above STh/L=  0.600        113 Note
PLAT913_ALERT_3_G Missing # of Very Strong Reflections in FCF ....          1 Note
PLAT933_ALERT_2_G Number of OMIT Records in Embedded .res File ...         23 Note
PLAT941_ALERT_3_G Average HKL Measurement Multiplicity ...........        2.7 Low
PLAT978_ALERT_2_G Number C-C Bonds with Positive Residual Density.         12 Info
```

---

0 **ALERT level A** = Most likely a serious problem - resolve or explain
0 **ALERT level B** = A potentially serious problem, consider carefully
5 **ALERT level C** = Check. Ensure it is not caused by an omission or oversight
10 **ALERT level G** = General information/check it is not something unexpected

1 ALERT type 1 CIF construction/syntax error, inconsistent or missing data
7 ALERT type 2 Indicator that the structure model may be wrong or deficient
4 ALERT type 3 Indicator that the structure quality may be low
2 ALERT type 4 Improvement, methodology, query or suggestion
1 ALERT type 5 Informative message, check

Datablock: 3b

Bond precision: C-C = 0.0016 A   Wavelength=0.71073

Cell:	a=10.0843(9)	b=11.4092(9)	c=10.8217(9)
	alpha=90	beta=95.443(8)	gamma=90
Temperature:	100 K		

	Calculated	Reported
Volume	1239.46(18)	1239.46(18)
Space group	P 21/n	P 1 21/n 1
Hall group	-P 2yn	-P 2yn
Moiety formula	C13 H15 N5 O2	C13 H15 N5 O2
Sum formula	C13 H15 N5 O2	C13 H15 N5 O2
Mr	273.30	273.30
Dx,g cm-3	1.465	1.465
Z	4	4
Mu (mm-1)	0.104	0.104
F000	576.0	576.0
F000’	576.22	
h,k,lmax	15,17,16	14,17,15
Nref	4412	4013
Tmin,Tmax	0.979,0.990	0.786,1.000
Tmin’	0.979	

Correction method= # Reported T Limits: Tmin=0.786 Tmax=1.000
AbsCorr = MULTI-SCAN

Data completeness= 0.910   Theta(max)= 32.300

R(reflections)= 0.0433(3303)   wR2(reflections)= 0.1126(4013)

S = 1.044   Npar= 176

The following ALERTS were generated. Each ALERT has the format
test-name_ALERT_alert-type_alert-level.
Click on the hyperlinks for more details of the test.

Alert level C

```
PLAT420_ALERT_2_C D-H Bond Without Acceptor  N5       --H5       .     Please Check
PLAT906_ALERT_3_C Large K Value in the Analysis of Variance ......      2.015 Check
PLAT911_ALERT_3_C Missing FCF Refl Between Thmin & STh/L=    0.600         20 Report
```

Alert level G

```
PLAT007_ALERT_5_G Number of Unrefined Donor-H Atoms ..............          1 Report
PLAT171_ALERT_4_G The CIF-Embedded .res File Contains EADP Records          1 Report
PLAT398_ALERT_2_G Deviating  C-O-C    Angle From 120 for O1             103.2 Degree
PLAT912_ALERT_4_G Missing # of FCF Reflections Above STh/L=  0.600        357 Note
PLAT933_ALERT_2_G Number of OMIT Records in Embedded .res File ...         19 Note
PLAT941_ALERT_3_G Average HKL Measurement Multiplicity ...........        3.5 Low
PLAT978_ALERT_2_G Number C-C Bonds with Positive Residual Density.          9 Info
```

0 **ALERT level A** = Most likely a serious problem - resolve or explain
0 **ALERT level B** = A potentially serious problem, consider carefully
3 **ALERT level C** = Check. Ensure it is not caused by an omission or oversight
7 **ALERT level G** = General information/check it is not something unexpected

0 ALERT type 1 CIF construction/syntax error, inconsistent or missing data
4 ALERT type 2 Indicator that the structure model may be wrong or deficient
3 ALERT type 3 Indicator that the structure quality may be low
2 ALERT type 4 Improvement, methodology, query or suggestion
1 ALERT type 5 Informative message, check

checkCIF publication errors

Alert level A

```
PUBL004_ALERT_1_A  The contact author's name and address are missing,
            _publ_contact_author_name and _publ_contact_author_address.
PUBL005_ALERT_1_A  _publ_contact_author_email, _publ_contact_author_fax and
            _publ_contact_author_phone are all missing.
            At least one of these should be present.
PUBL006_ALERT_1_A  _publ_requested_journal is missing
            e.g. 'Acta Crystallographica Section C'
PUBL008_ALERT_1_A  _publ_section_title is missing. Title of paper.
PUBL009_ALERT_1_A  _publ_author_name is missing. List of author(s) name(s).
PUBL010_ALERT_1_A  _publ_author_address is missing. Author(s) address(es).
PUBL012_ALERT_1_A  _publ_section_abstract is missing.
            Abstract of paper in English.
```

7 **ALERT level A** = Data missing that is essential or data in wrong format
0 **ALERT level G** = General alerts. Data that may be required is missing

Publication of your CIF

You should attempt to resolve as many as possible of the alerts in all categories. Often the minor alerts point to easily fixed oversights, errors and omissions in your CIF or refinement strategy, so attention to these fine details can be worthwhile. In order to resolve some of the more serious problems it may be necessary to carry out additional measurements or structure refinements. However, the nature of your study may justify the reported deviations from journal submission requirements and the more serious of these should be commented upon in the discussion or experimental section of a paper or in the "special_details" fields of the CIF. *checkCIF* was carefully designed to identify outliers and unusual parameters, but every test has its limitations and alerts that are not important in a particular case may appear. Conversely, the absence of alerts does not guarantee there are no aspects of the results needing attention. It is up to the individual to critically assess their own results and, if necessary, seek expert advice.

If level A alerts remain, which you believe to be justified deviations, and you intend to submit this CIF for publication in a journal, you should additionally insert an explanation in your CIF using the Validation Reply Form (VRF) below. This will allow your explanation to be considered as part of the review process.

Validation response form

Please find below a validation response form (VRF) that can be filled in and pasted into your CIF.

```
# start Validation Reply Form
_vrf_PUBL004_GLOBAL
;
PROBLEM: The contact author’s name and address are missing,
RESPONSE: ...
;
_vrf_PUBL005_GLOBAL
;
PROBLEM: _publ_contact_author_email, _publ_contact_author_fax and
RESPONSE: ...
;
_vrf_PUBL006_GLOBAL
;
PROBLEM: _publ_requested_journal is missing
RESPONSE: ...
;
_vrf_PUBL008_GLOBAL
;
PROBLEM: _publ_section_title is missing. Title of paper.
RESPONSE: ...
;
_vrf_PUBL009_GLOBAL
;
PROBLEM: _publ_author_name is missing. List of author(s) name(s).
RESPONSE: ...
;
_vrf_PUBL010_GLOBAL
;
PROBLEM: _publ_author_address is missing. Author(s) address(es).
RESPONSE: ...
;
_vrf_PUBL012_GLOBAL
;
PROBLEM: _publ_section_abstract is missing.
```

```
RESPONSE: ...
;
_vrf_PLAT906_1a
;
PROBLEM: Large K Value in the Analysis of Variance ......      3.001 Check
RESPONSE: ...
;
_vrf_PLAT911_1a
;
PROBLEM: Missing FCF Refl Between Thmin & STh/L=    0.600          7 Report
RESPONSE: ...
;
_vrf_PLAT913_1a
;
PROBLEM: Missing # of Very Strong Reflections in FCF ....          4 Note
RESPONSE: ...
;
_vrf_PLAT250_1b
;
PROBLEM: Large U3/U1 Ratio for Average U(i,j) Tensor ....        2.6 Note
RESPONSE: ...
;
_vrf_PLAT906_2a
;
PROBLEM: Large K Value in the Analysis of Variance ......      6.995 Check
RESPONSE: ...
;
_vrf_PLAT911_2a
;
PROBLEM: Missing FCF Refl Between Thmin & STh/L=    0.600          9 Report
RESPONSE: ...
;
_vrf_PLAT241_3a
;
PROBLEM: High   'MainMol' Ueq as Compared to Neighbors of        N1A Check
RESPONSE: ...
;
_vrf_PLAT250_3a
;
PROBLEM: Large U3/U1 Ratio for Average U(i,j) Tensor ....        2.4 Note
RESPONSE: ...
;
_vrf_PLAT417_3a
;
PROBLEM: Short Inter D-H..H-D       H4AA     ..H3B      .       2.10 Ang.
RESPONSE: ...
;
_vrf_PLAT906_3a
;
PROBLEM: Large K Value in the Analysis of Variance ......      5.563 Check
RESPONSE: ...
;
_vrf_PLAT911_3a
;
PROBLEM: Missing FCF Refl Between Thmin & STh/L=    0.600         11 Report
RESPONSE: ...
;
_vrf_PLAT420_3b
;
PROBLEM: D-H Bond Without Acceptor  N5       --H5       .     Please Check
RESPONSE: ...
;
```

```
_vrf_PLAT906_3b
;
PROBLEM: Large K Value in the Analysis of Variance ......      2.015 Check
RESPONSE: ...
;
_vrf_PLAT911_3b
;
PROBLEM: Missing FCF Refl Between Thmin & STh/L=    0.600         20 Report
RESPONSE: ...
;
# end Validation Reply Form
```

If you wish to submit your CIF for publication in Acta Crystallographica Section C or E, you should upload your CIF via the web. If you wish to submit your CIF for publication in IUCrData you should upload your CIF via the web. If your CIF is to form part of a submission to another IUCr journal, you will be asked, either during electronic submission or by the Co-editor handling your paper, to upload your CIF via our web site.

PLATON version of 03/06/2021; check.def file version of 02/06/2021

Datablock 1a - ellipsoid plot

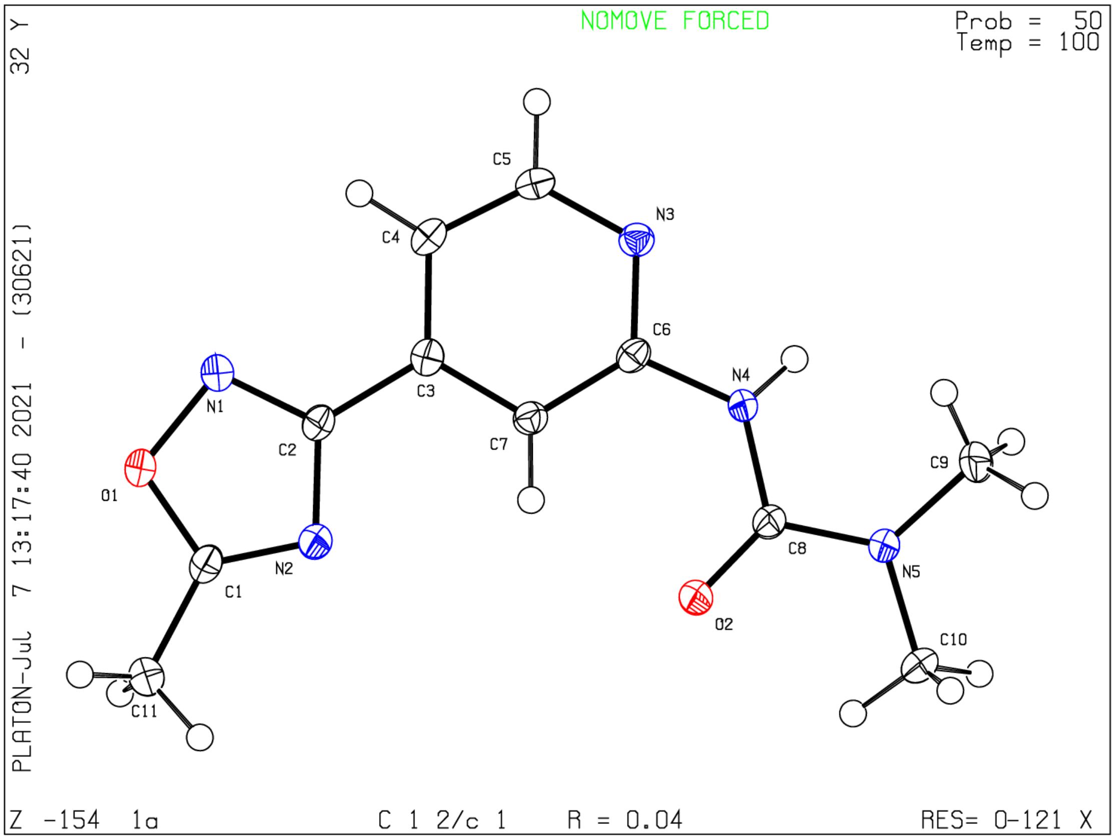

Datablock 1b - ellipsoid plot

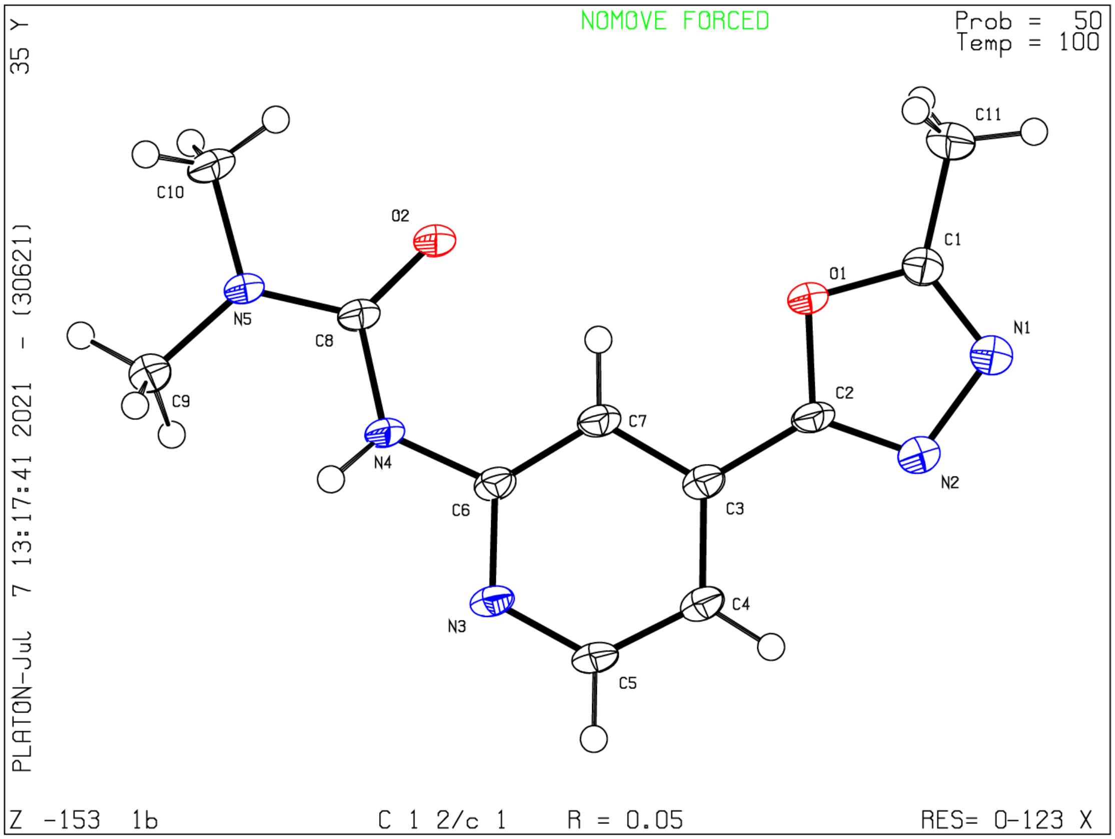

Datablock 2a - ellipsoid plot

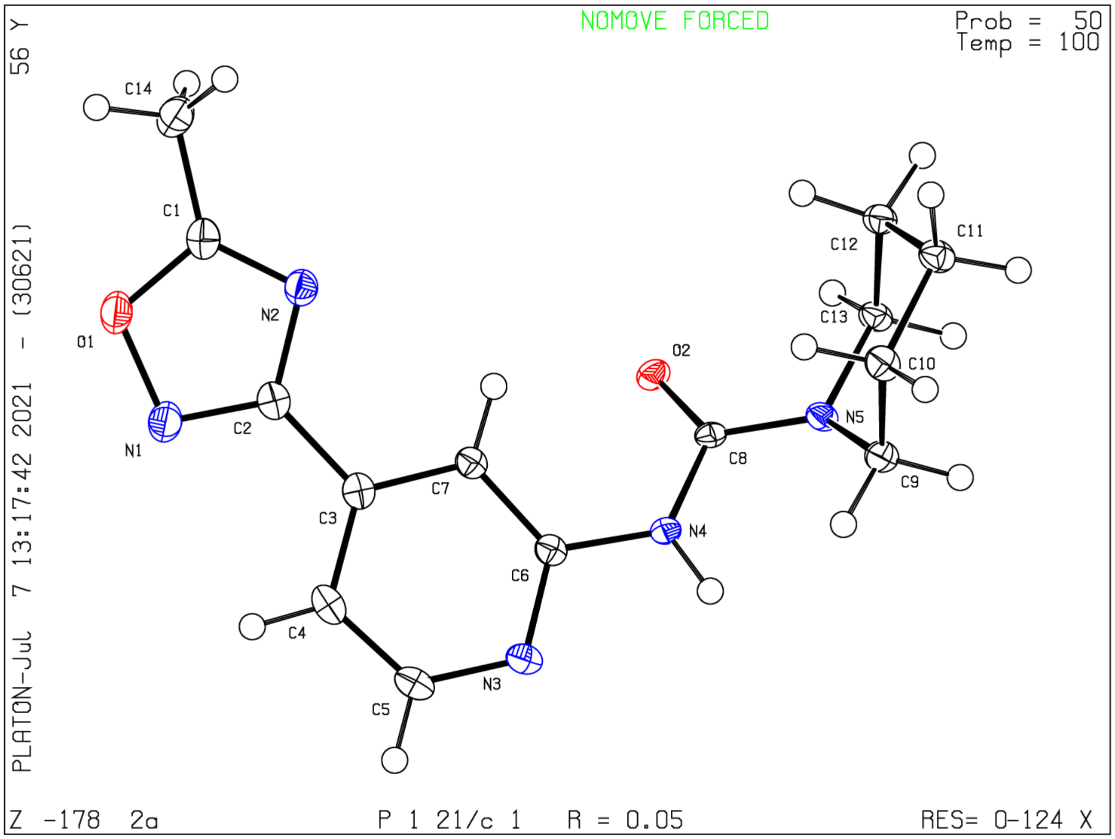

Datablock 2b - ellipsoid plot

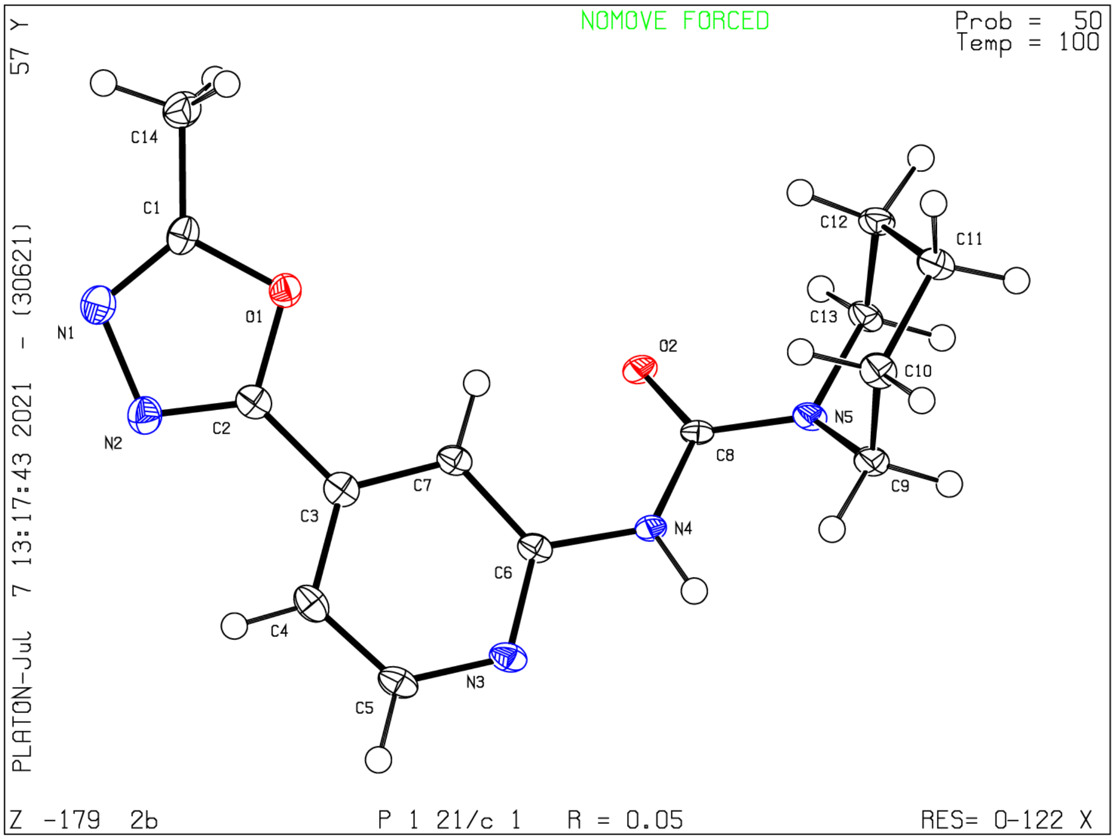

Datablock 3a - ellipsoid plot

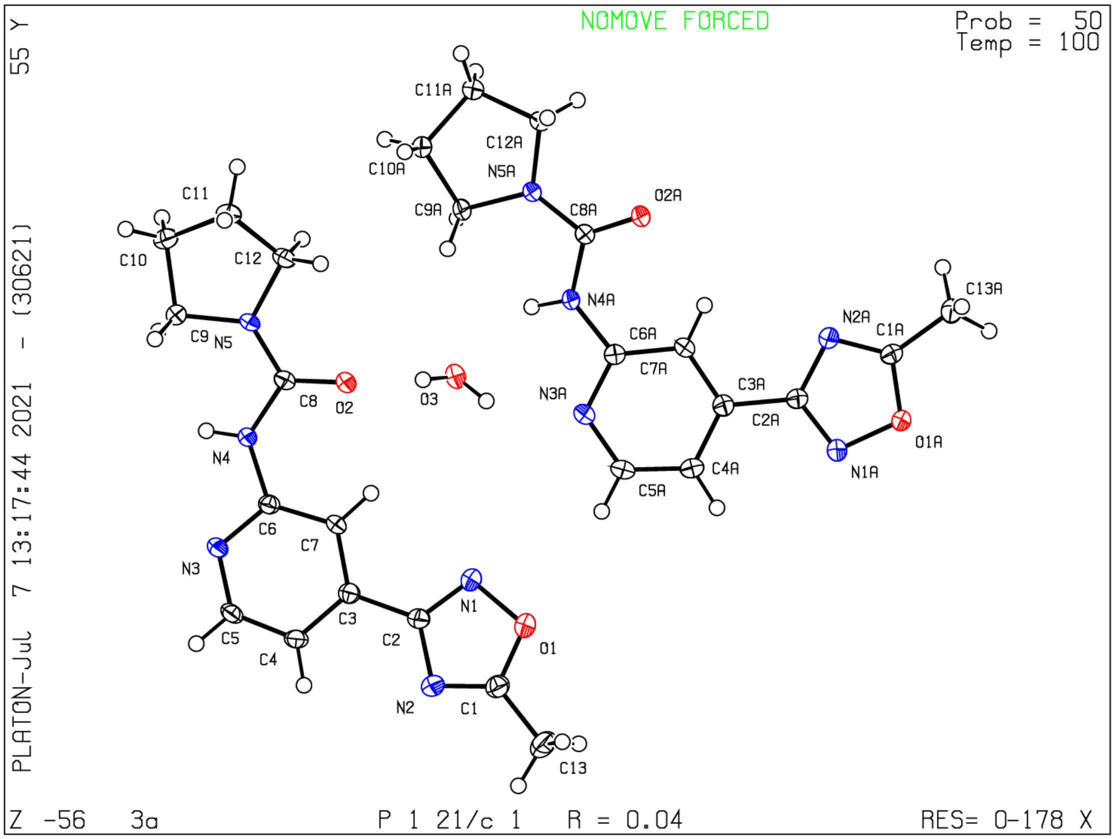

Datablock 3b - ellipsoid plot

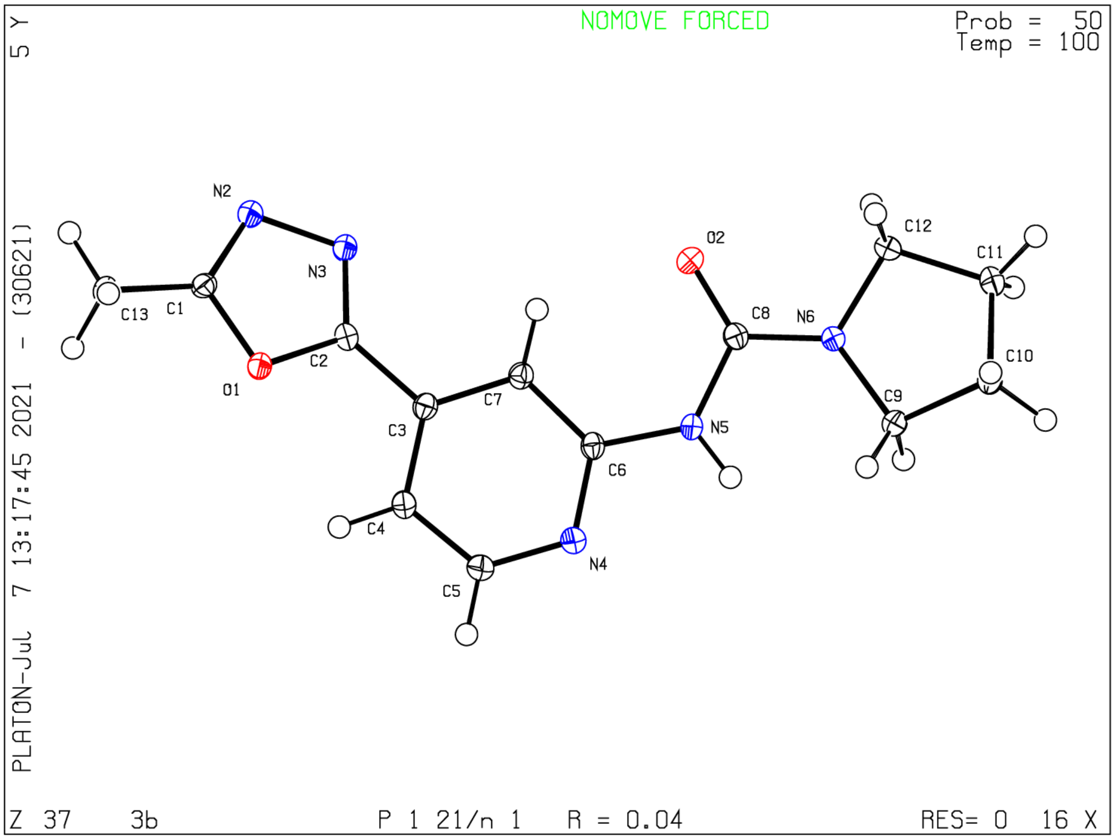

Supplement: Supplementary file 1 [file molecules-26-05672-s001.zip › 1-3a,b_checkcif.pdf]
