# Supplementary material for: π–π Noncovalent Interaction Involving 1,2,4- and 1,3,4-Oxadiazole Systems: The Combined Experimental, Theoretical, and Database Study
Source: Molecules. 2021 Sep 18;26(18):5672. doi: 10.3390/molecules26185672 (PMC8466036; doi:10.3390/molecules26185672)
Supplement: Supplementary file 1 [file molecules-26-05672-s001.zip › Supporting Information.pdf]

Supporting Information

for

π-π Noncovalent Interaction Involving 1,2,4- and 1,3,4-Oxadiazole Systems: The Combined Experimental, Theoretical, and Database Study

Sergey V. Baykov,[a] Alexander S. Mikherdov,[a] Alexander S. Novikov,[a] Kirill K. Geyl,[a] Marina V. Tarasenko,[b] Maxim A. Gureev,[c] Vadim P. Boyarskiy[a]*

[a]Institute of Chemistry, Saint Petersburg State University, 7/9 Universitetskaya Nab., Saint Petersburg 199034, Russian Federation

[b]Pharmaceutical Technology Transfer Centre, Yaroslavl State Pedagogical University named after K.D. Ushinsky, 108 Respublikanskaya St., 150000 Yaroslavl, Russian Federation

[c]Research Center "Digital biodesign and personalized healthcare", I.M. Sechenov First Moscow State Medical University, 119991 Moscow, Russian Federation

* Correspondence: v.boiarskii@spbu.ru

Content

S1. Crystallographic information 3

S2. Synthetic scheme and NMR spectra of *N*-pyridinylureas.. 9

S3. Calculation details 13

S4. Supramolecular association in solution 21

S5. 3GO1 model cluster preparation.. 24

S6. References.. 26

S1. Crystallographic information

Table S1. Crystal data and structure refinement for **1a**, **1b**, and **2a**.			
Compound	**1a**	**1b**	**2a**
Identification code	bs-403-1	BSC–065	bsc-201
CCDC number	1903615	1903620	2093232
Empirical formula	$C_{11}H_{13}N_5O_2$	$C_{11}H_{13}N_5O_2$	$C_{14}H_{17}N_5O_2$
Formula weight	247.26	247.26	287.32
Temperature/K	100(2)	100(2)	100(2)
Crystal system	monoclinic	monoclinic	monoclinic
Space group	C2/c	C2/c	$P2_1/c$
a/Å	20.4216(11)	20.2667(8)	15.4284(8)
b/Å	4.8213(2)	4.8772(2)	9.4251(5)
c/Å	25.8398(15)	25.3025(9)	9.5049(4)
α/°	90	90	90
β/°	111.743(7)	109.643(4)	91.712(4)
γ/°	90	90	90
Volume/Å^3	2363.1(2)	2355.47(17)	1381.53(12)
Z	8	8	4
ρ_{calc} g/cm^3	1.390	1.395	1.381
μ/mm^{-1}	0.101	0.839	0.097
F(000)	1040.0	1040.0	608.0
Crystal size/mm^3	0.2 × 0.18 × 0.1	0.16 × 0.15 × 0.11	0.18 × 0.13 × 0.13
Radiation	Mo Kα (λ = 0.71073)	Cu Kα (λ = 1.54184)	Mo Kα (λ = 0.71073)
2Θ range for data collection/°	5.17 to 53.998	7.42 to 144.842	6.088 to 64.728
Index ranges	$-25 \le h \le 26$, $-6 \le k \le 6$, $-33 \le l \le 30$	$-24 \le h \le 24$, $-5 \le k \le 6$, $-31 \le l \le 31$	$-23 \le h \le 20$, $-13 \le k \le 13$, $-14 \le l \le 14$
Reflections collected	10355	24114	10429
Independent reflections	2566 [R_{int} = 0.0244, R_{sigma} = 0.0227]	2308 [R_{int} = 0.1014, R_{sigma} = 0.0401]	4391 [R_{int} = 0.0301, R_{sigma} = 0.0477]
Data/restraints/parameters	2566/1/171	2308/1/170	4391/0/195
Goodness-of-fit on F^2	1.055	1.030	1.057
Final R indexes [I≥2σ (I)]	R_1 = 0.0421, wR_2 = 0.1017	R_1 = 0.0484, wR_2 = 0.1287	R_1 = 0.0528, wR_2 = 0.1049
Final R indexes [all data]	R_1 = 0.0462, wR_2 = 0.1044	R_1 = 0.0556, wR_2 = 0.1370	R_1 = 0.0735, wR_2 = 0.1151
Largest diff. peak/hole / e·Å^{-3}	0.26/–0.2	0.22/–0.30	0.36/–0.28

**Table S2**. Crystal data and structure refinement for **2b**, **3a**, and **3b**.			
Compound	**2b**	**3a**	**3b**
Identification code	bsc-202	bsc-203	bsc-205
CCDC number	2093234	2093238	2093239
Empirical formula	$C_{14}H_{17}N_5O_2$	$C_{26}H_{32}N_{10}O_5$	$C_{13}H_{15}N_5O_2$
Formula weight	287.32	564.61	273.30
Temperature/K	100(2)	100(2)	100(2)
Crystal system	monoclinic	monoclinic	monoclinic
Space group	$P2_1/c$	$P2_1/c$	$P2_1/n$
a/Å	15.1212(2)	8.4559(2)	10.0843(9)
b/Å	9.51420(10)	13.9116(3)	11.4092(9)
c/Å	9.52360(10)	23.0116(5)	10.8217(9)
α/°	90	90	90
β/°	91.8210(10)	93.125(2)	95.443(8)
γ/°	90	90	90
Volume/Å^3	1369.43(3)	2702.95(10)	1239.46(18)
Z	4	4	4
ρ_{calc} g/cm^3	1.394	1.387	1.465
μ/mm^{-1}	0.801	0.830	0.104
F(000)	608.0	1192.0	576.0
Crystal size/mm^3	0.17 × 0.12 × 0.12	0.19 × 0.13 × 0.12	0.16 × 0.15 × 0.15
Radiation	Cu Kα (λ = 1.54184)	Cu Kα (λ = 1.54184)	Mo Kα (λ = 0.71073)
2Θ range for data collection/°	5.848 to 147.868	7.428 to 152.204	5.2 to 64.6
Index ranges	–17 ≤ h ≤ 18, –11 ≤ k ≤ 11, –10 ≤ l ≤ 11	–10 ≤ h ≤ 7, –17 ≤ k ≤ 17, –25 ≤ l ≤ 28	–14 ≤ h ≤ 14, –17 ≤ k ≤ 16, –15 ≤ l ≤ 15
Reflections collected	8225	14946	14024
Independent reflections	2754 [R_{int} = 0.0282, R_{sigma} = 0.0270]	5538 [R_{int} = 0.0351, R_{sigma} = 0.0356]	4013 [R_{int} = 0.0320, R_{sigma} = 0.0338]
Data/restraints/parameters	2754/0/195	5538/0/375	4013/0/176
Goodness-of-fit on F^2	1.037	1.039	1.044
Final R indexes [I≥2σ (I)]	R_1 = 0.0467, wR_2 = 0.1282	R_1 = 0.0438, wR_2 = 0.1099	R_1 = 0.0433, wR_2 = 0.1044
Final R indexes [all data]	R_1 = 0.0488, wR_2 = 0.1303	R_1 = 0.0589, wR_2 = 0.1175	R_1 = 0.0556, wR_2 = 0.1126
Largest diff. peak/hole / e·Å^{-3}	0.27/–0.44	0.34/–0.35	0.49/–0.35

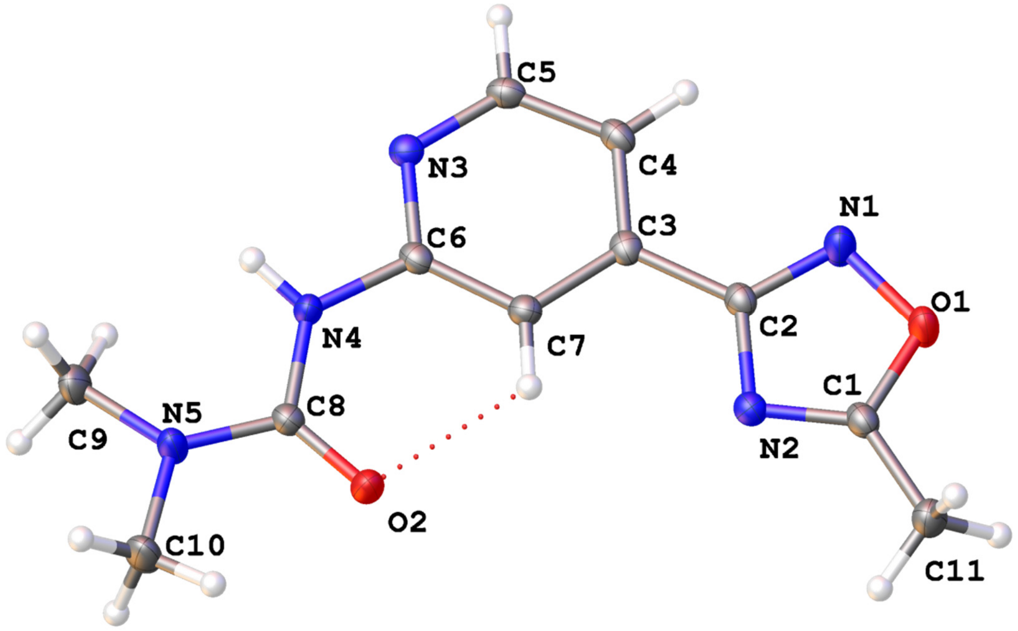

Figure S1. Structure of **1a**.

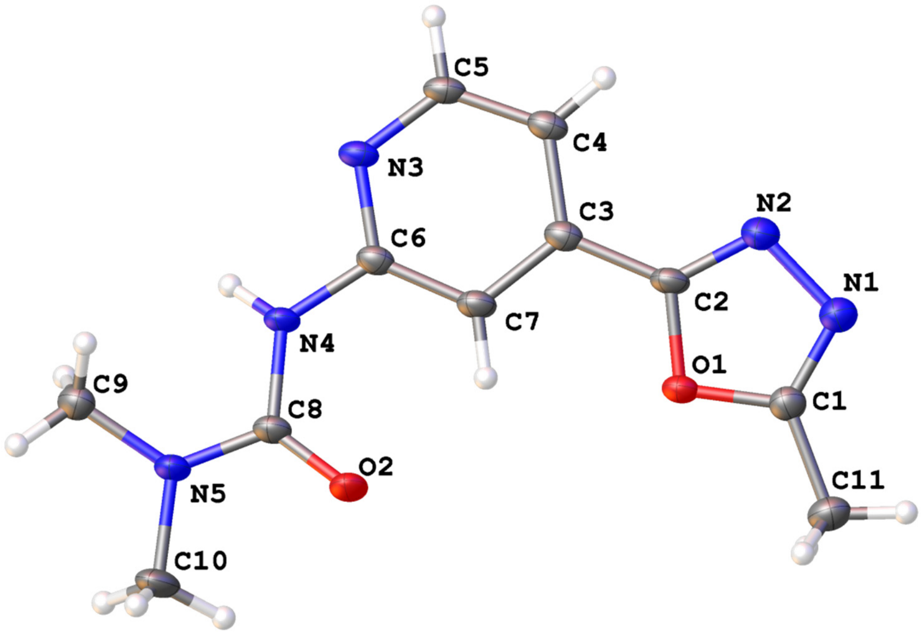

Figure S2. Structure of **1b**.

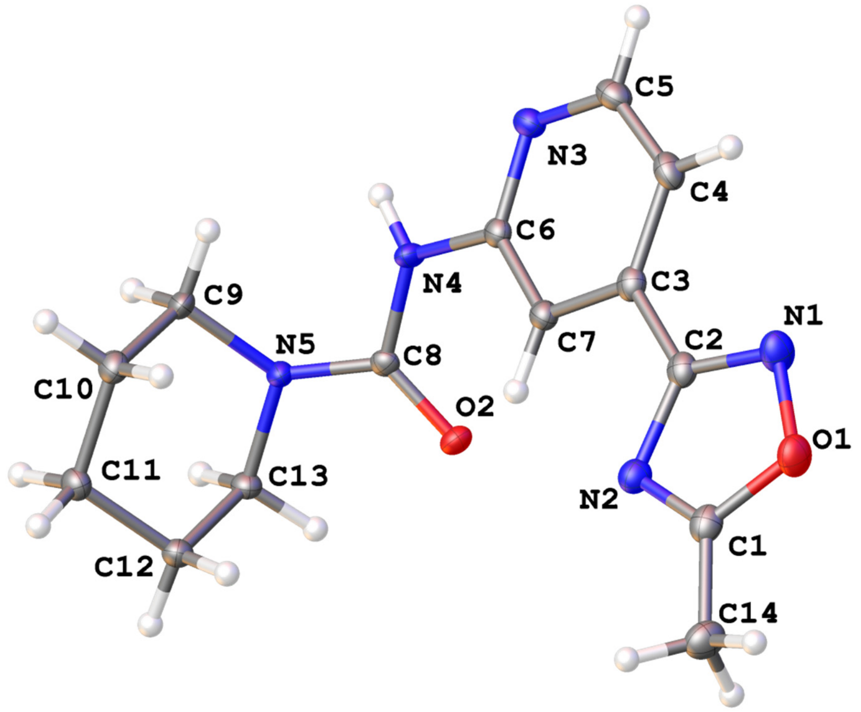

Figure S3. Structure of **2a**.

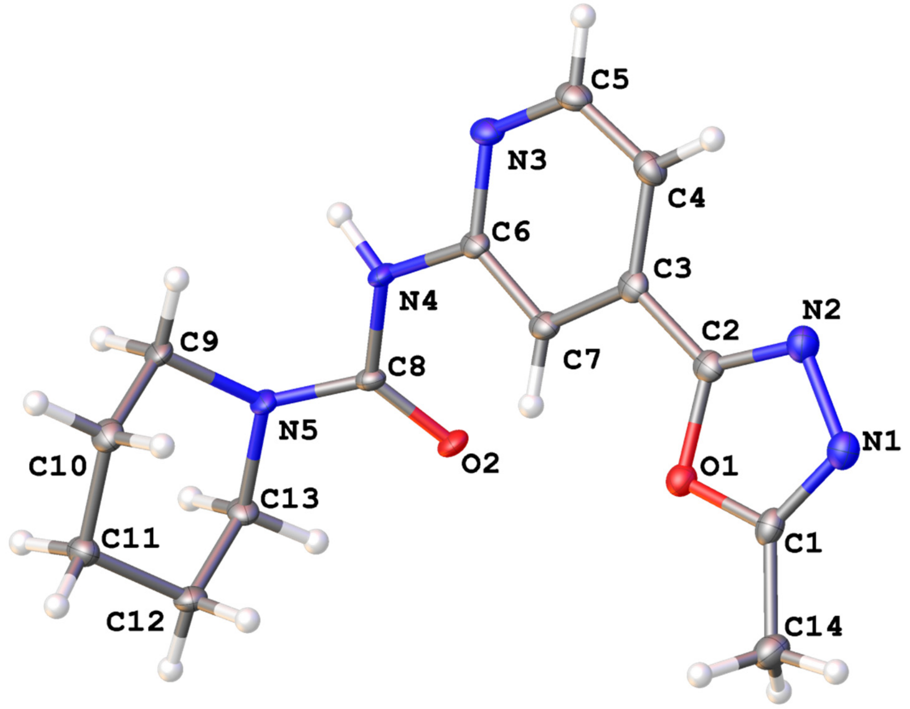

Figure S4. Structure of **2b**.

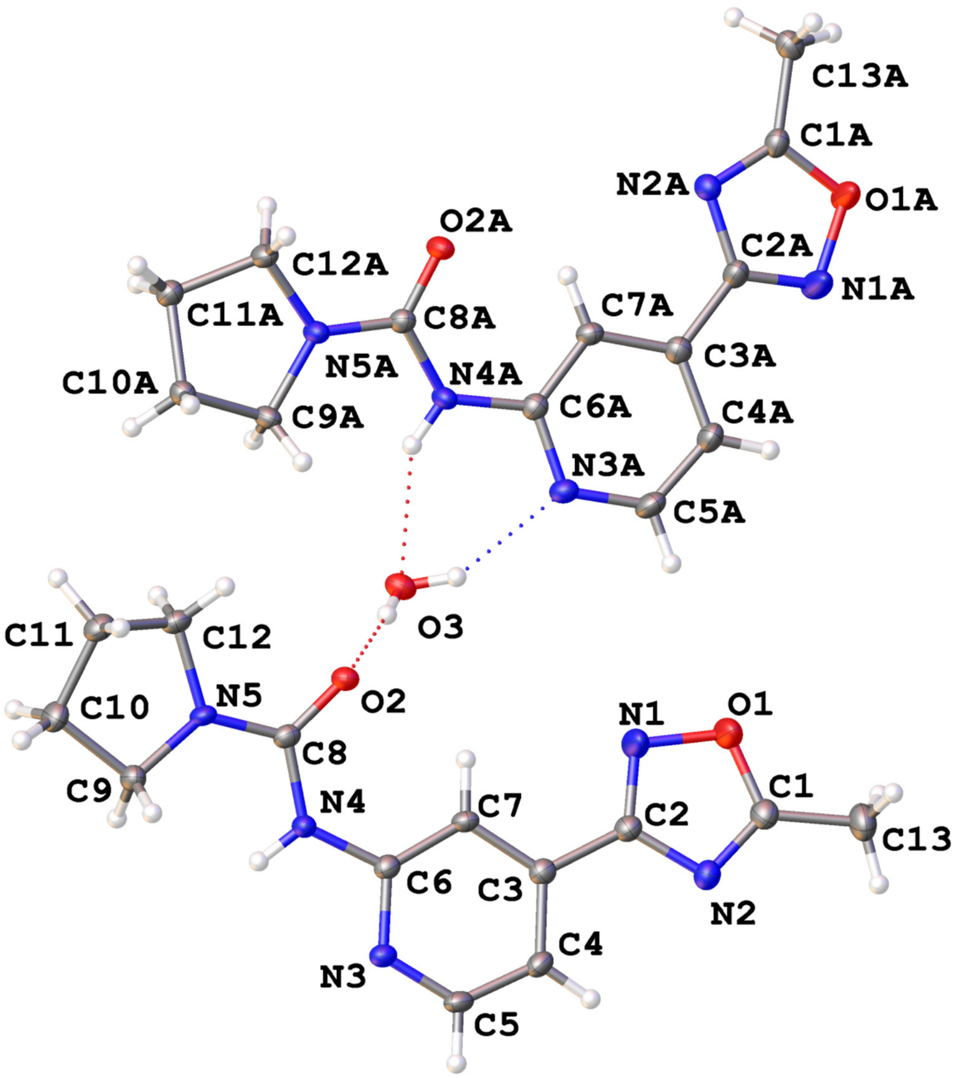

Figure S5. Structure of **3a**.

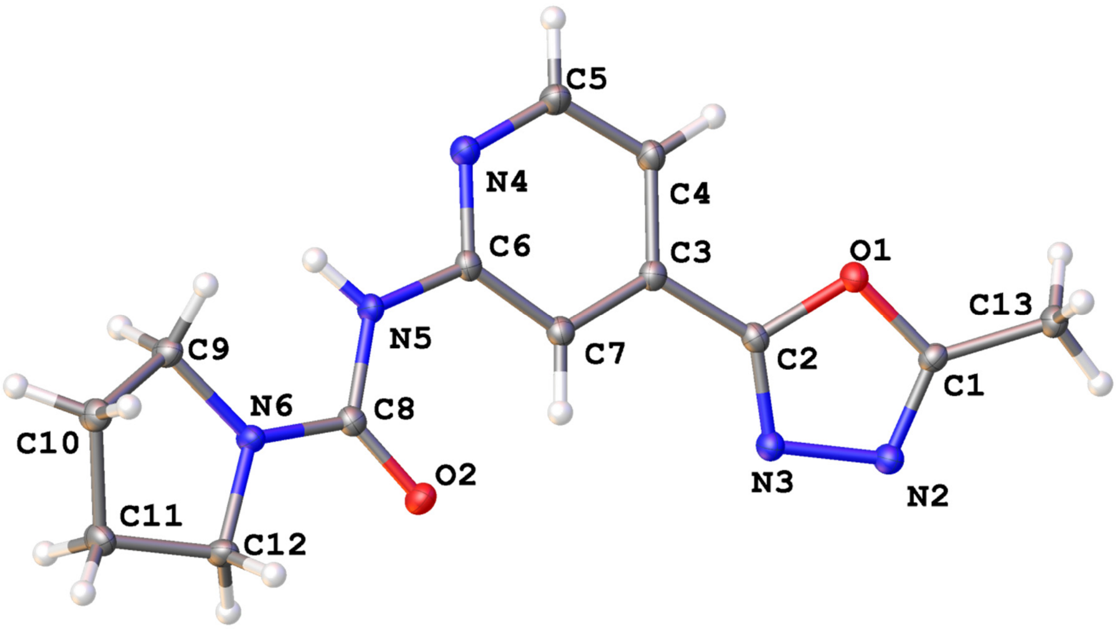

Figure S6. Structure of **3b**.

S2. Synthetic scheme and NMR spectra of *N*-pyridinylureas

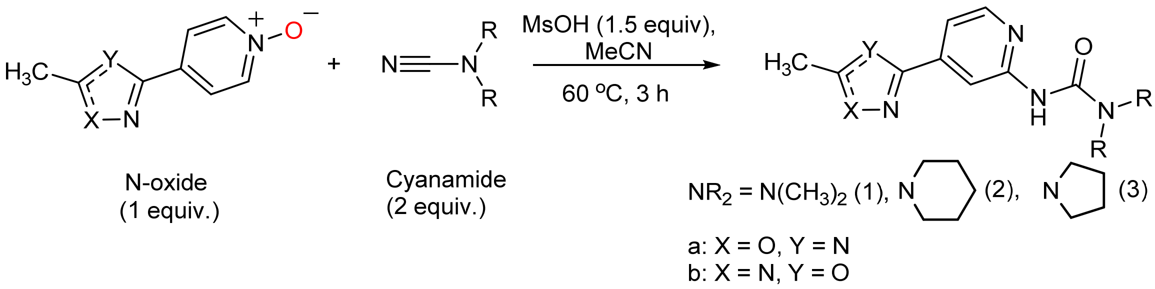

Scheme S1. Synthesis of *N*-pyridyl ureas bearing oxadiazole moiety.

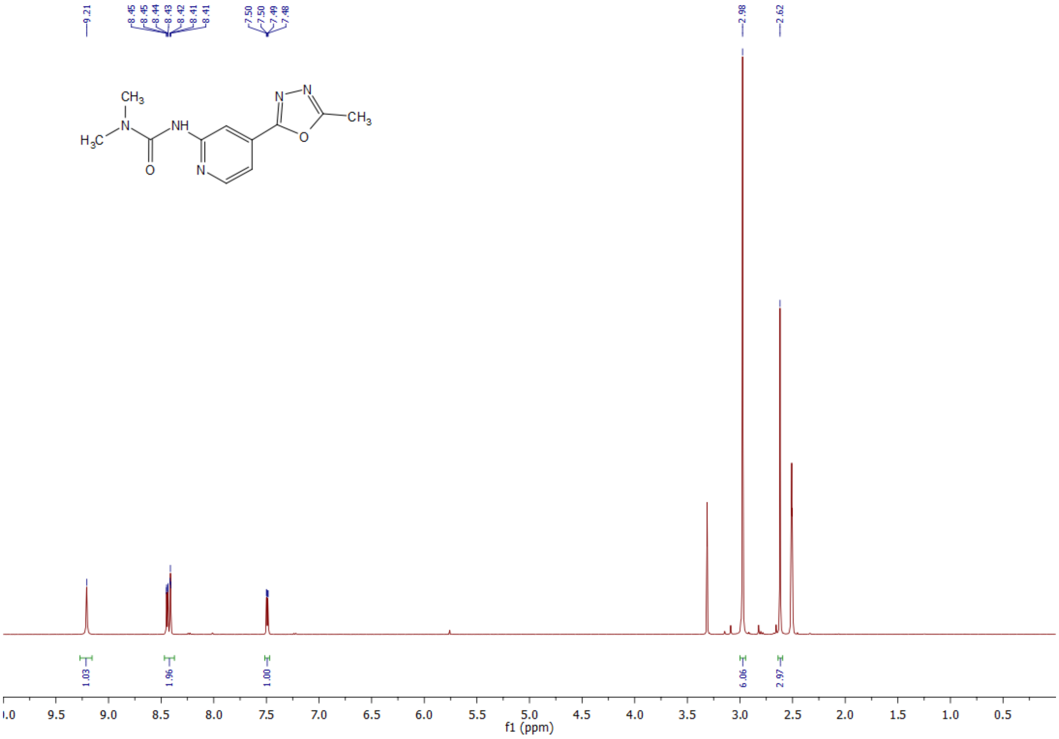

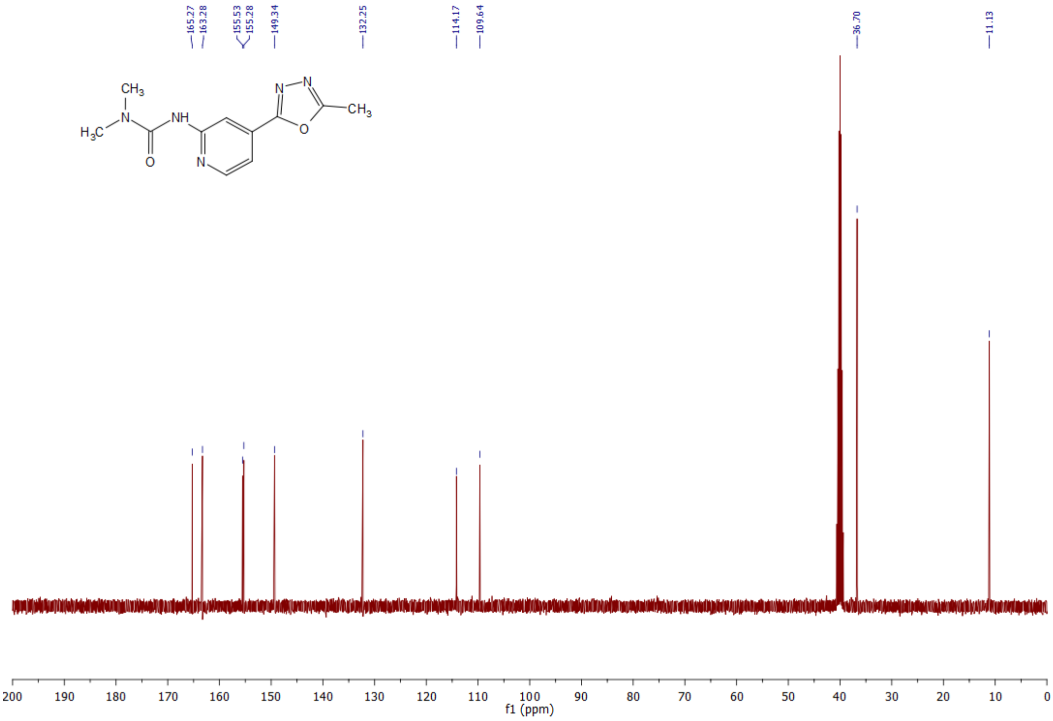

Figure S7. ^{1}H and ^{13}C NMR spectra 1,1-dimethyl-3-(4-(5-methyl-1,3,4-oxadiazol-2-yl)pyridin-2-yl)urea **1a**.

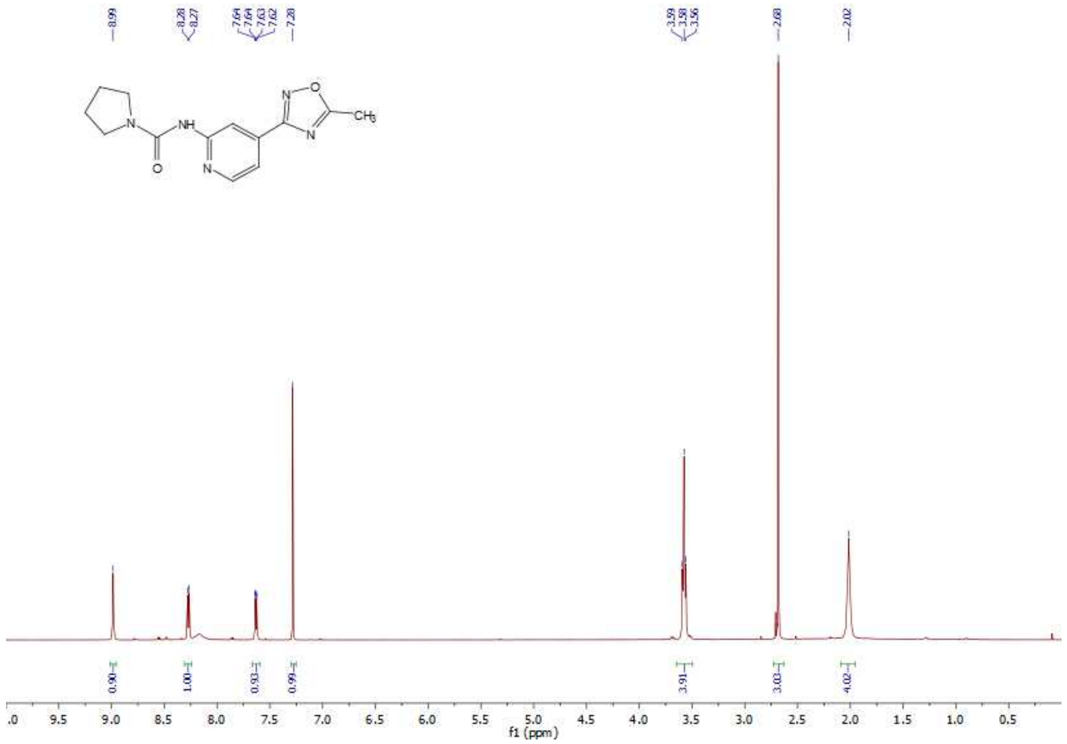

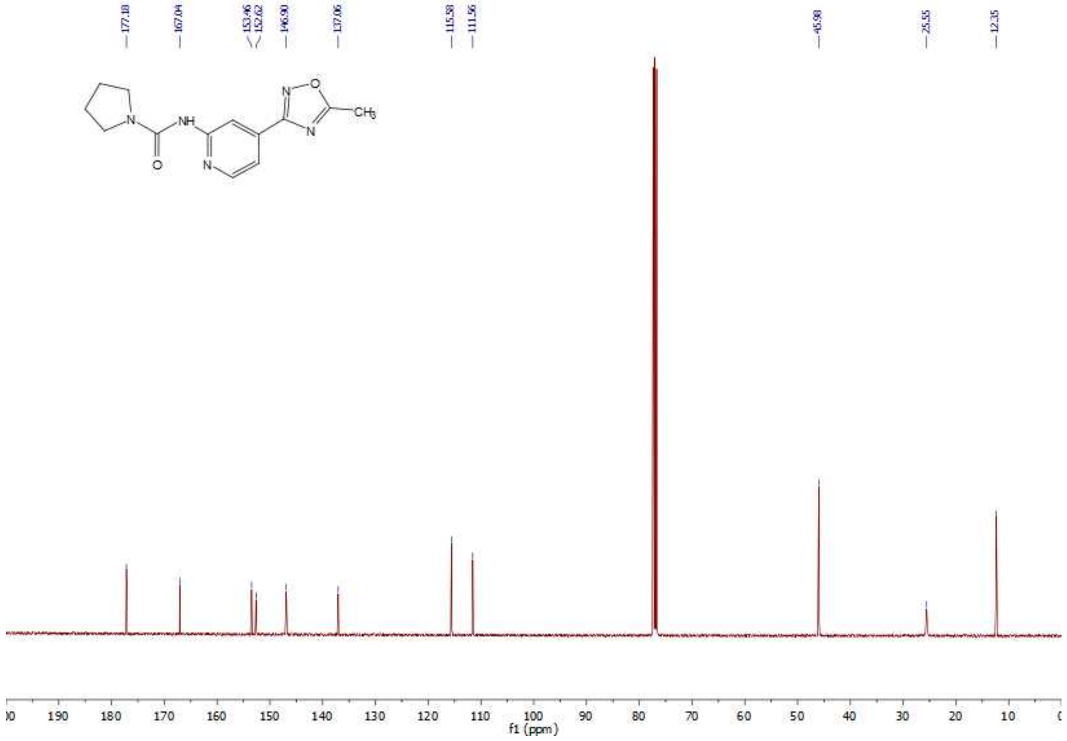

Figure S8. ^{1}H and ^{13}C NMR spectra *N*-(4-(5-Methyl-1,2,4-oxadiazol-3-yl)pyridin-2-yl)pyrrolidine-1-carboxamide **3a**

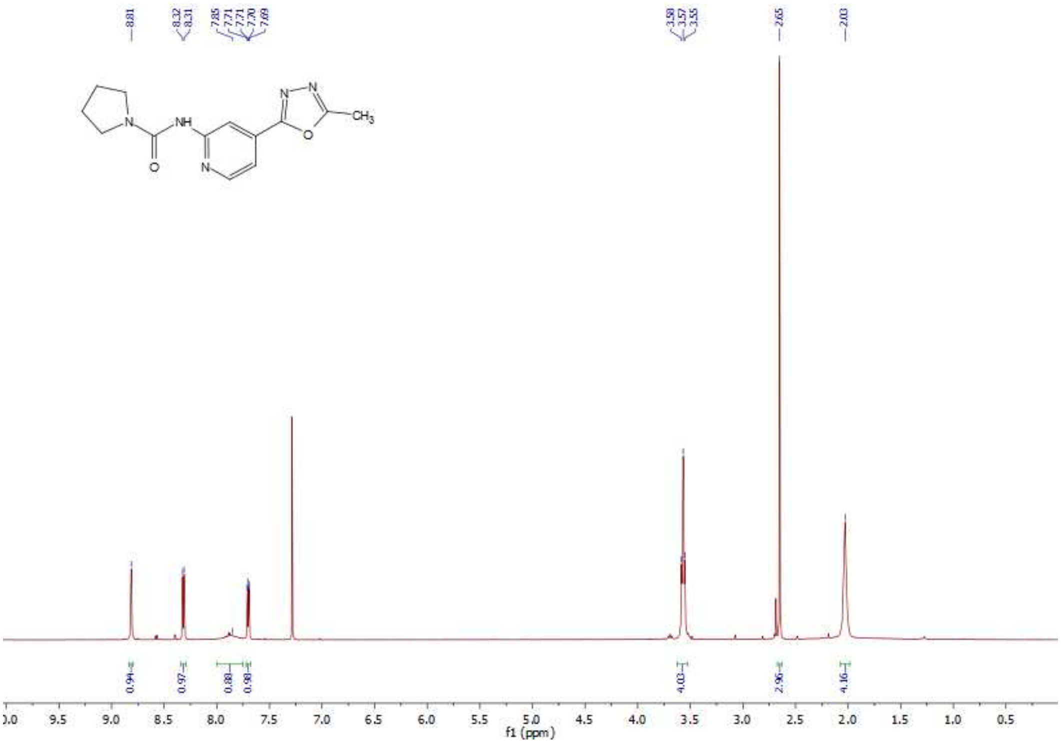

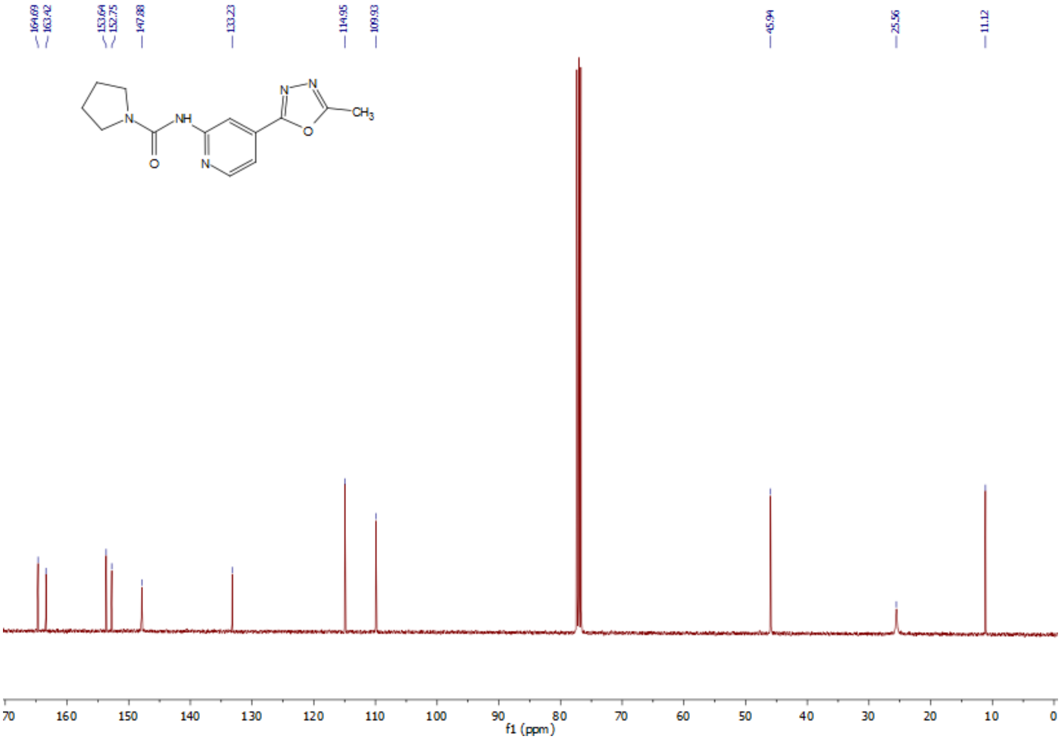

Figure S9. ^{1}H and ^{13}C NMR spectra *N*-(4-(5-Methyl-1,3,4-oxadiazol-2-yl)pyridin-2-yl)pyrrolidine-1-carboxamide **3b**

S3. Calculation details

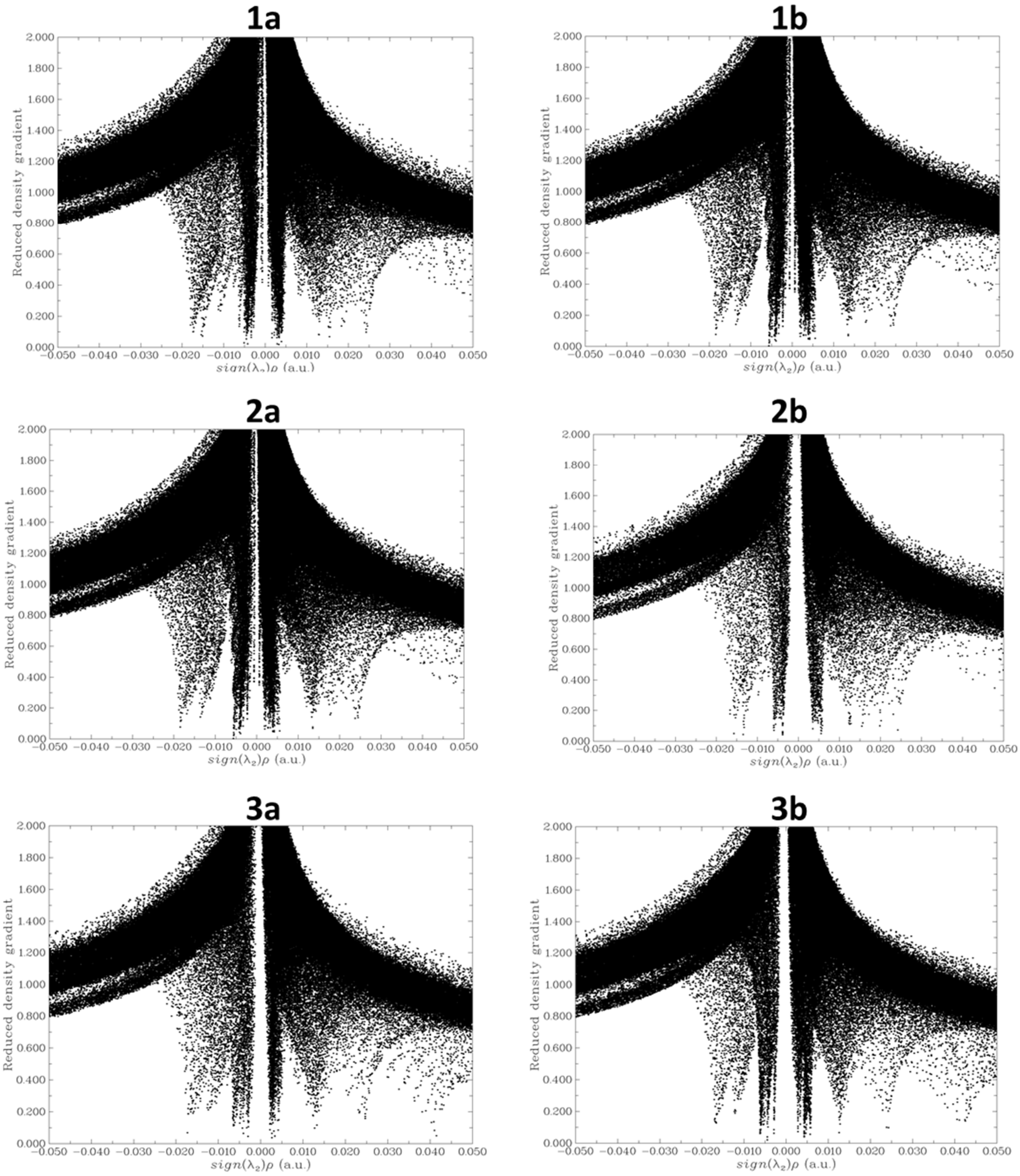

Figure S10. NCI plots for model dimeric associates of **(1–3)a,b**.

Optimization of model dimeric associates (1–3)a,b (ωB97XD/6-31G* level of theory)

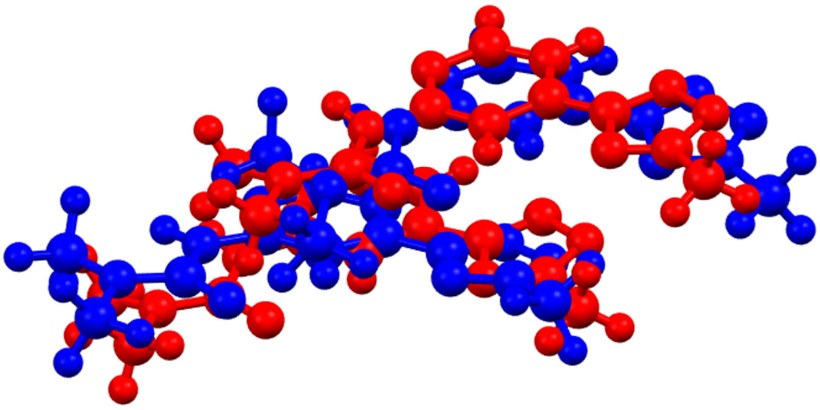

Figure S11. Overlapped experimental XRD (red) and theoretically optimized (ωB97XD/6-31G*; blue) structures of dimeric associate of **1a**.

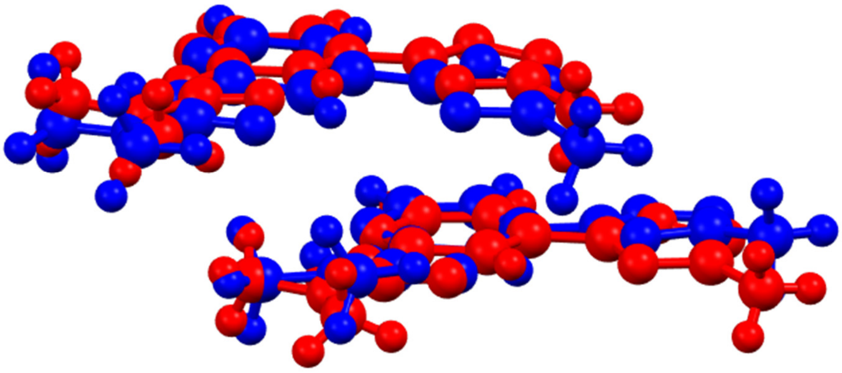

Figure S12. Overlapped experimental XRD (red) and theoretically optimized (ωB97XD/6-31G*; blue) structures of dimeric associate of **1b**.

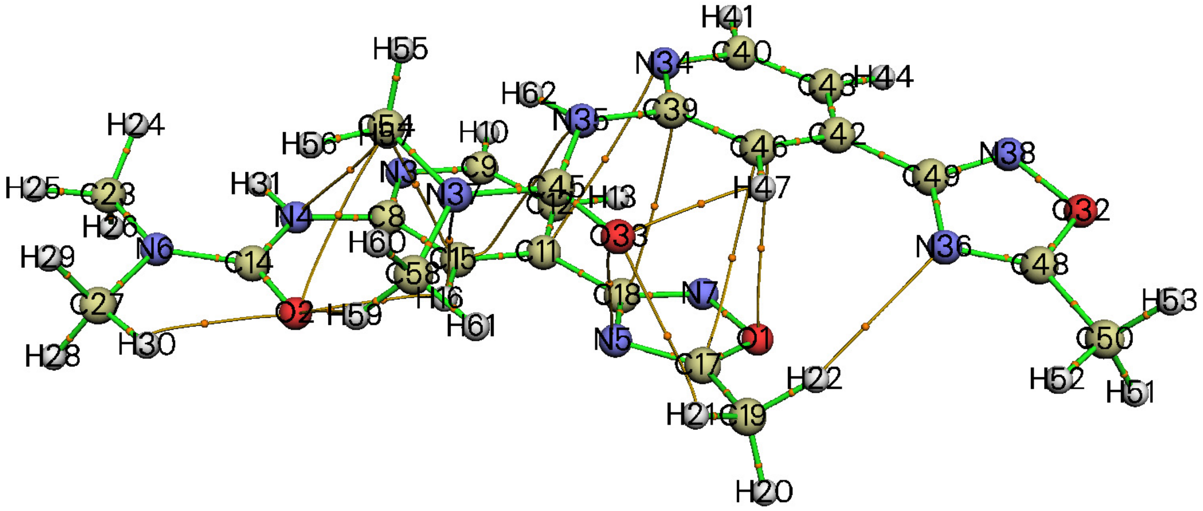

Figure S13. Molecular graph from the QTAIM analysis of the optimized structure of model dimeric associate of **1a** (ωB97XD/6-31G*). Bond critical points (3, −1) are shown in orange. The Poincaré–Hopf relationship is satisfied.

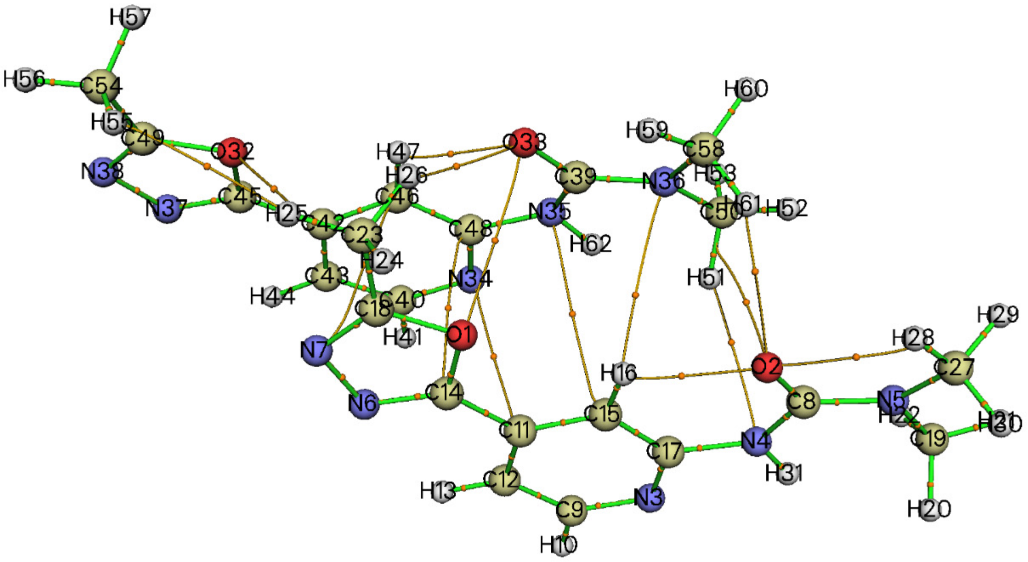

Figure S14. Molecular graph from the QTAIM analysis of the optimized structure of model dimeric associate of **1b** (ωB97XD/6-31G*). Bond critical points (3, −1) are shown in orange. The Poincaré–Hopf relationship is satisfied.

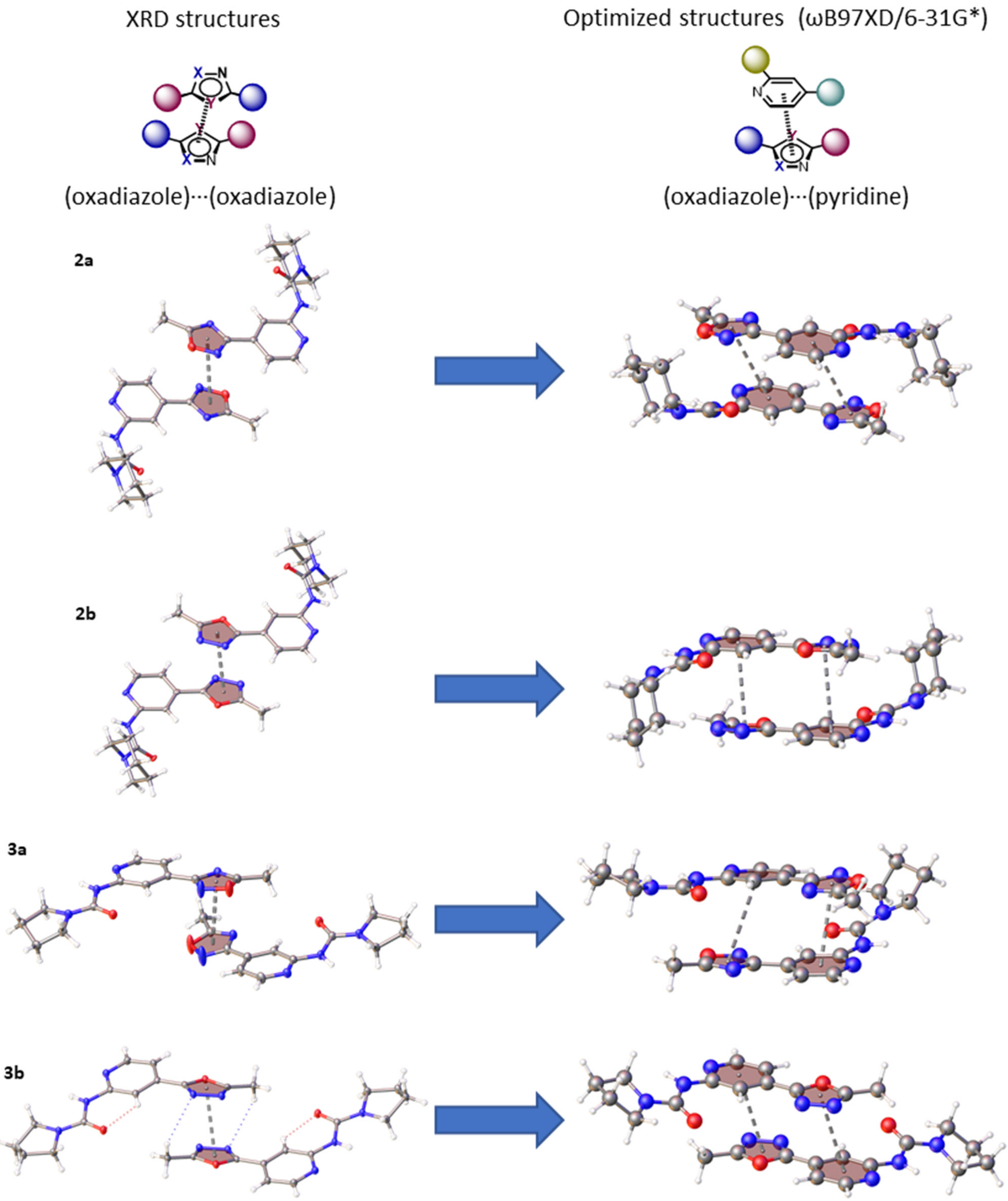

Figure S15. Experimental XRD (left) and theoretically optimized structures (ωB97XD/6-31G*; right) of dimeric associates of **(2–3)a,b**.

Table S3. The distances (Å) between centroids of oxadiazole and pyridine rings in the experimental and theoretical structures of **(1–3)a,b**

Structure	Experimental		Optimized	
	d(Oxa-Oxa) Å	d(Oxa-Py)	d(Oxa-Oxa)	d(Oxa-Py)
**1a**	4.821	3.623	5.000	3.446
**1b**	4.877	3.520	4.615	3.458
**2a**	3.684	5.027	6.299	3.575
**2b**	3.485	4.913	4.771	3.958
**3a**	3.502	5.995	5.507	4.313
**3b**	3.398	5.698	4.193	3.523

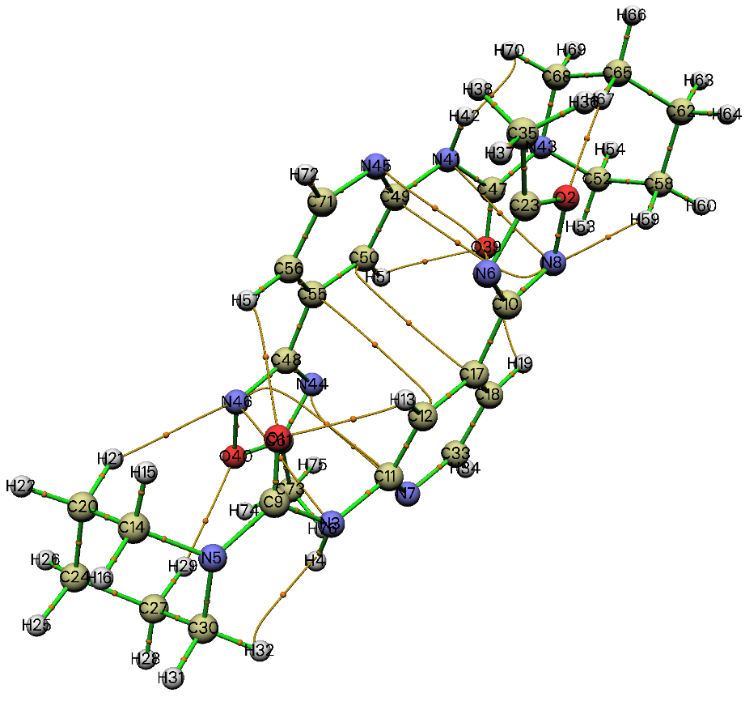

Figure S16. Molecular graph from the QTAIM analysis of the optimized structure of model dimeric associate of **2a** (ωB97XD/6-31G*). Bond critical points (3, −1) are shown in orange. The Poincaré–Hopf relationship is satisfied.

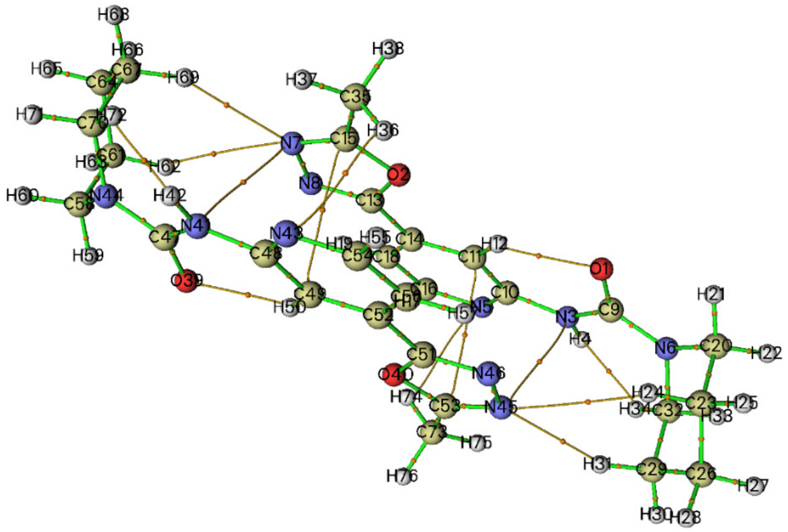

Figure S17. Molecular graph from the QTAIM analysis of the optimized structure of model dimeric associate of **2b** (ωB97XD/6-31G*). Bond critical points (3, −1) are shown in orange. The Poincaré–Hopf relationship is satisfied.

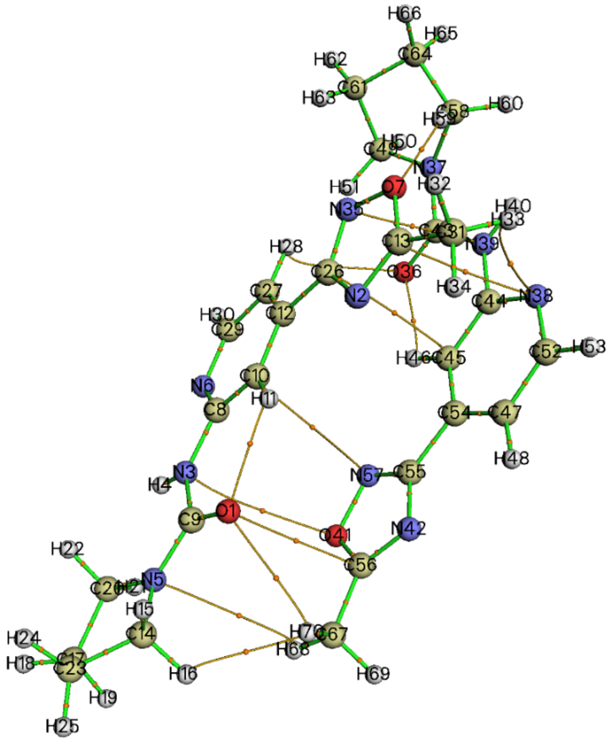

Figure S18. Molecular graph from the QTAIM analysis of the optimized structure of model dimeric associate of **3a** (ωB97XD/6-31G*). Bond critical points (3, −1) are shown in orange. The Poincaré–Hopf relationship is satisfied.

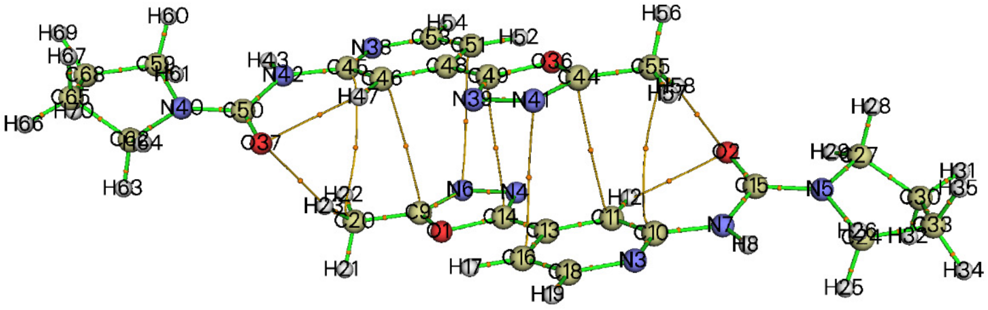

Figure S19. Molecular graph from the QTAIM analysis of the optimized structure of model dimeric associate of **3b** (ωB97XD/6-31G*). Bond critical points (3, −1) are shown in orange. The Poincaré–Hopf relationship is satisfied.

Table S4. Values of the density of all electrons – ρ(**r**), Laplacian of electron density – ∇^2ρ(**r**), energy density – H_b, potential energy density – V(**r**), and Lagrangian kinetic energy – G(**r**) (a.u.) at the bond critical points (3, –1), and interatomic distance – *l* (Å), corresponding to stacking interactions in optimized equilibrium geometries of model dimeric associates (**1**–**3**)**a**,**b** (ωB97XD/6-31G*) and **1a,b** (ωB97XD/6-311+G*).†

Contacts	ρ(**r**)	∇^2ρ(**r**)	H_b	V(**r**)	G(**r**)	*l**
			**1a**(ωB97XD/6-31G*)			
C46···O1	0.007	0.023	0.001	-0.004	0.005	3.256
C46···C17	0.007	0.023	0.001	-0.004	0.005	3.227
C39···C18	0.006	0.022	0.001	-0.003	0.004	3.239
N5···O33	0.008	0.028	0.001	-0.006	0.006	3.100
			**1a(**ωB97XD/6-311+G*)			
C46···O1	0.006	0.021	0.001	-0.004	0.004	3.286
C46···C17	0.006	0.022	0.001	-0.003	0.004	3.254
C39···C18	0.006	0.021	0.001	-0.003	0.004	3.269
N5···O33	0.008	0.027	0.001	-0.005	0.006	3.130
			**1b**(ωB97XD/6-31G*)			
O1···O33	0.007	0.027	0.001	-0.006	0.006	3.055
N7···C46	0.007	0.022	0.001	-0.004	0.005	3.326
C14···C48	0.006	0.021	0.001	-0.003	0.004	3.235
			**1b(**ωB97XD/6-311+G*)			
O1···O33	0.007	0.025	0.001	-0.005	0.006	3.083
N7···C46	0.007	0.021	0.001	-0.003	0.004	3.359
C14···C48	0.006	0.021	0.001	-0.003	0.004	3.263
			**2a**(ωB97XD/6-31G*)			
N8···C49	0.006	0.020	0.001	-0.003	0.004	3.303
N8···N41	0.007	0.022	0.000	-0.005	0.005	3.236
N6···N45	0.008	0.023	0.001	-0.005	0.005	3.223
			**2b**(ωB97XD/6-31G*)			
N45···N3	0.005	0.018	0.001	-0.003	0.004	3.399
C53···C11	0.007	0.022	0.001	-0.003	0.004	3.249
			**3a**(ωB97XD/6-31G*)			
N57···C10	0.007	0.019	0.001	-0.004	0.004	3.276
O41···N3	0.005	0.020	0.001	-0.004	0.004	3.299
C56···O1	0.007	0.028	0.001	-0.005	0.006	3.057
			**3b**(ωB97XD/6-31G*)			
C9···C46	0.006	0.018	0.001	-0.003	0.004	3.332
N6···C51	0.006	0.020	0.001	-0.004	0.004	3.310
C14···C49	0.006	0.019	0.001	-0.003	0.004	3.287

† Poincare-Hopf relationship was satisfied.

* The shortest van der Waals radii for N, C, and O atoms are 1.55, 1.70, and 1.52 Å, respectively [1].

S4. Supramolecular association in solution

A series of diffusion coefficient (D) measurements by NMR spectroscopy were performed for sample **1a** in $CDCl_3$ (concentrations ranging from 611 mM down to 6 mM for **1a**). The D values show good sensitivity to the effective size of supramolecular associates, and a degree of association can be approximately estimated using the Stokes−Einstein equation [2–4].

$$D = \frac{\mathrm{kT}}{6\pi\eta r_H} \quad (1)$$

where k is Boltzmann constant, η is the viscosity of the solvent, and r_H is the hydrodynamic radius.

Under the conditions of a fast exchange between monomeric and any associated forms of **1a** the observed diffusion coefficient (D_{obs}) is an average of the species presented in solution weighted with their relative amount.

Eq. 1 is valid for the spherical objects, the size of which is exceedingly larger than that of the solvent. Therefore, the r_H values of elongated nonspherical molecules calculated using the Stokes−Einstein equation is a rough approach. Nevertheless, the dependence of an average $r_{H(av)}$ value (derived from D_{obs}) on concentration is an indicative assessment of the association degree, as it points to the average number of individual units within the associates.

To eliminate the influence of variable viscosity (η) at different concentrations on the D_{obs}, tetramethylsilane (TMS) was used as an internal standard which not subject to association and value D_{TMS}/D_{obs} were used for the further calculation. Because TMS is not subject to the association and its r_H is constant, the ratio D_{TMS}/D_{obs} is also proportional to $r_{H(av)}$ of the associate. The aggregation number N is defined as:

$$N = \frac{V_H}{V_H^0} = \left(\frac{r_{H(av)}}{r_H^0}\right)^3 = \left(\frac{D_0}{D_{obs}}\right)^3 \quad (2)$$

where V_H and V_H^0, are the hydrodynamic volumes of the observed associate and that of the monomer, r_H^0 – the hydrodynamic radius of the monomer, D_0 is corresponds to the formal absence of aggregation at infinite dilution, obtained by extrapolating the D_{TMS}/D_{obs} to zero concentration of **1a** and is equivalent to the diffusion coefficient of the monomer (D_M) (**Figure**

S20). The calculated average aggregation numbers (N), D_{obs}, and D_{TMS}/D_{obs} values are given in **Table S5**.

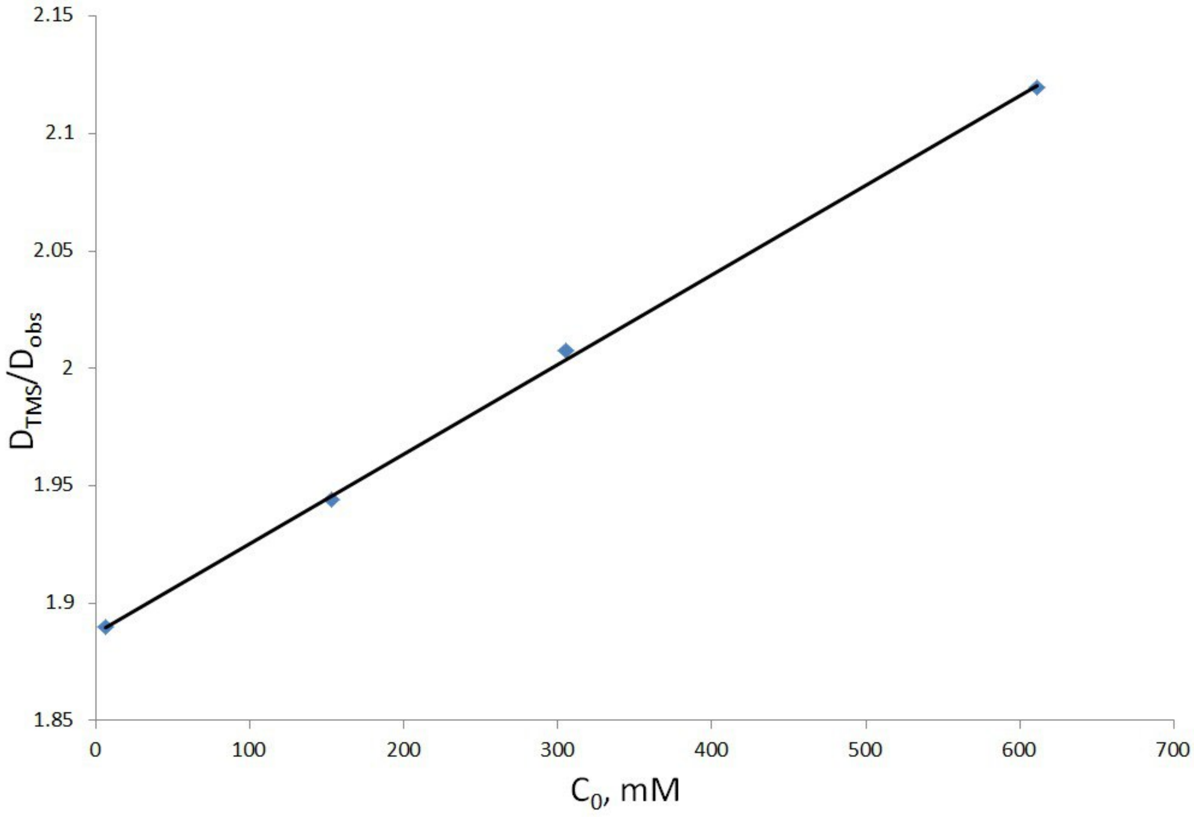

Figure S20. Dependence of the D_{TMS}/D_{obs} ratio on the concentration of **1a** in $CDCl_3$ at 298 K.

Table S5. Observed and corrected diffusion coefficients (D_{obs} and D_{TMS}/D_{obs}) and average aggregation numbers (N) for **1a** in $CDCl_3$ at 298 K.

C_o, mM	D_{obs}, $\mu m^2 s^{-1}$	D_{TMS}/D_{obs}	N
0[a]	-	1.89	1.00
6	995	1.89	1.00
153	890	1.94	1.09
306	802	2.01	1.20
611	604	2.12	1.42

[a] extrapolation.

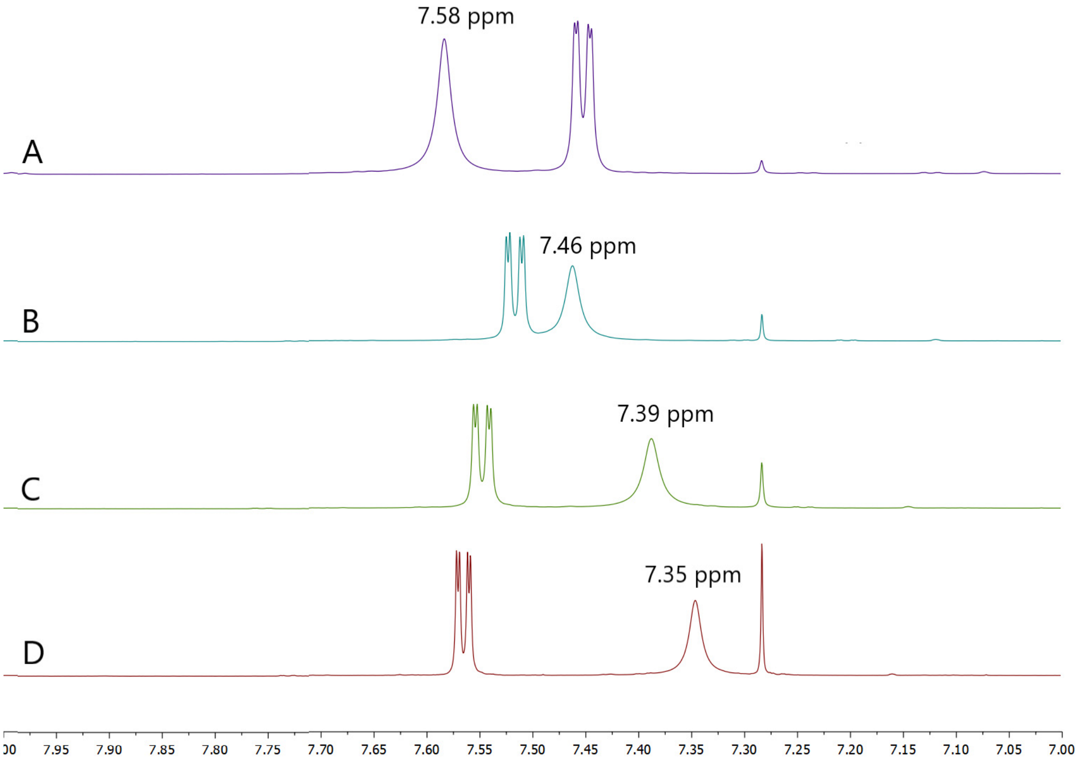

Figure S21. Chemical shits of NH proton in ^{1}H NMR spectra of **1a** ($CDCl_3$) depending on sample concentration (A – 611 mM, B – 306 mM, C – 153 mM, D – 76 mM).

Table S6. Selected ^{1}H and ^{13}C signals of **1a** at 75 mM and 611 mM in $CDCl_3$

Atom	Concentration of **1a**	
	75 mM	611 mM
	^{13}C chemical shift, ppm	
C1	177.05	177.05
C2	167.22	167.13
C3	136.33	136.16
C4	111.25	111.25
C5	153.77	153.87
C6	148.21	148.11
C7	115.78	115.62
CO	154.59	154.69
NCH3	36.42	36.38
CH3	12.34	12.28
	^{1}H chemical shift, ppm	
NH	7.37	7.58

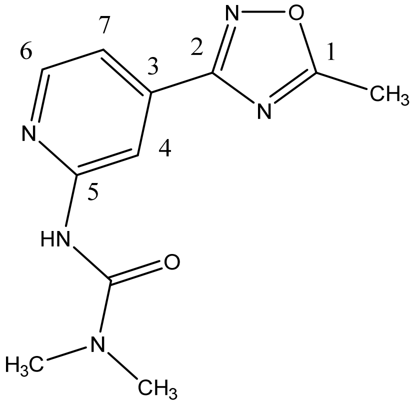

S5. 3GO1 model cluster preparation

1. *Transcriptional repressor EthR structure preparation:*

Receptor structure was downloaded from RCSB Protein data bank, ID **3G1O**. The raw structure, obtained by the NMR method and have some mismatches in the protein model: missing hydrogens and incorrect bond orders. Also, the observed EthR model contains water molecules.

Our EthR structure was prepared with the use of Protein PrepWizard [5] (Schrodinger Suite 2020-4): assigned bond orders, missing hydrogens and sidechains added, water molecules are deleted from protein-ligand complex (they do not form bridge interactions entire ligand/protein). Also, our structure was refined by restrained minimization in OPLS3e [6] forcefield, to heavy atom RMSD equal to 0.30Å.

2. *Active site cluster preparation:*

Selected ligand from observed EthR model (PDB ID 3G1O) is included in a workspace with surrounding residues in 4 Å radius from the ligand. All selected atoms were extracted in a separate entry. The main point of our interest is the core-containing moiety – oxadiazole scaffold of our compound.

Oxadiazole scaffold is located between Phe110, Trp145, and Asn176/179. Principal intermolecular contacts of oxadiazole core are realized with Phe110. This residue forms energetically favorable hydrophobic contact supported by π-π stacking of aromatic systems. Asn179 gives hydrogen bond to nitrogen, non-linked with oxygen in oxadiazole ring.

In case of full model cluster 3G1O (Figure S21), it is difficult to select one dominated region on the scatter graph of reduced density gradient (RDG) vs. real space function sign(λ_2)ρ (NCI plot). Therefore, the reduced cluster 3GO1 for NCI analysis was prepared by omitting of all fragments except the oxadiazole scaffold and interacting with it Phe110 and carbonyl moieties.

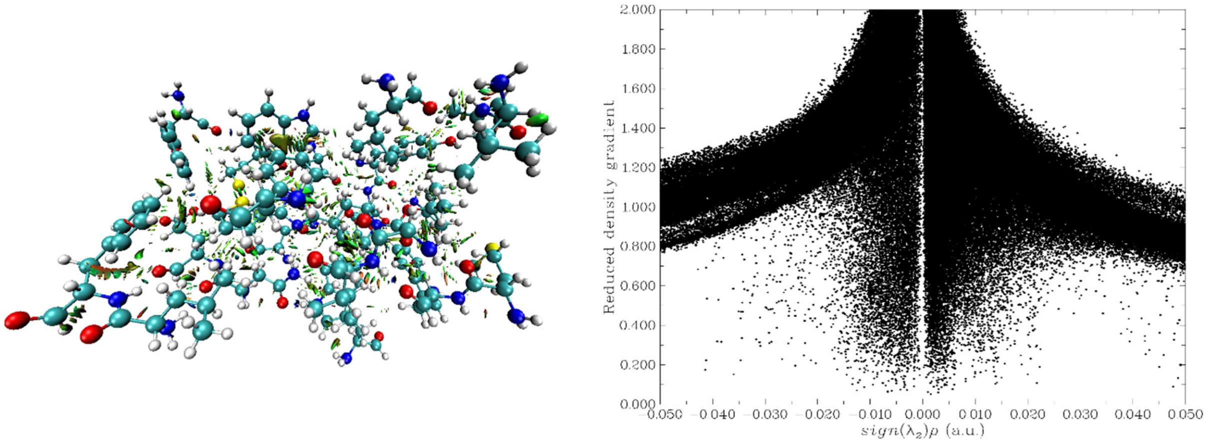

Figure S21. NCI plot for full model cluster 3G1O and visualization of various noncovalent interactions in 3D using NCI analysis technique.

S6. References

[1] A. Bondi, van der Waals Volumes and Radii, J. Phys. Chem. 68 (1964) 441–451. doi:10.1021/j100785a001.

[2] V. V. Sivchik, E. V. Grachova, A.S. Melnikov, S.N. Smirnov, A.Y. Ivanov, P. Hirva, S.P. Tunik, I.O. Koshevoy, Solid-State and Solution Metallophilic Aggregation of a Cationic [Pt(NCN)L] + Cyclometalated Complex, Inorg. Chem. 55 (2016) 3351–3363. doi:10.1021/acs.inorgchem.5b02713.

[3] G. Bellachioma, G. Ciancaleoni, C. Zuccaccia, D. Zuccaccia, A. Macchioni, NMR investigation of non-covalent aggregation of coordination compounds ranging from dimers and ion pairs up to nano-aggregates, Coord. Chem. Rev. 252 (2008) 2224–2238. doi:10.1016/j.ccr.2007.12.016.

[4] A. Macchioni, G. Ciancaleoni, C. Zuccaccia, D. Zuccaccia, Determining accurate molecular sizes in solution through NMR diffusion spectroscopy, Chem. Soc. Rev. 37 (2008) 479–489. doi:10.1039/B615067P.

[5] G. Madhavi Sastry, M. Adzhigirey, T. Day, R. Annabhimoju, W. Sherman, Protein and ligand preparation: parameters, protocols, and influence on virtual screening enrichments, J. Comput. Aided. Mol. Des. 27 (2013) 221–234. doi:10.1007/s10822-013-9644-8.

[6] K. Roos, C. Wu, W. Damm, M. Reboul, J.M. Stevenson, C. Lu, M.K. Dahlgren, S. Mondal, W. Chen, L. Wang, R. Abel, R.A. Friesner, E.D. Harder, OPLS3e: Extending Force Field Coverage for Drug-Like Small Molecules, J. Chem. Theory Comput. 15 (2019) 1863–1874. doi:10.1021/acs.jctc.8b01026.
